# Modular Polymerase Synthesis and Internal Protein Domain Swapping via Dual Opposed Frameshifts in the Ebola Virus L Gene

**DOI:** 10.3390/pathogens13100829

**Published:** 2024-09-25

**Authors:** David B. Stubbs, Jan A. Ruzicka, Ethan W. Taylor

**Affiliations:** Department of Chemistry and Biochemistry, University of North Carolina at Greensboro, P.O. Box 26170, Greensboro, NC 27402-6170, USA; dbstubbs1993@gmail.com (D.B.S.); jaruzick@uncg.edu (J.A.R.)

**Keywords:** antisense, deiodinase II, ebolavirus, mRNA, ribosomal frameshift, selenium, selenoprotein

## Abstract

Sequence analysis of the Zaire ebolavirus (EBOV) polymerase (L gene) mRNA, using online tools, identified a highly ranked −1 programmed ribosomal frameshift (FS) signal including an ideal slippery sequence heptamer (UUUAAAA), with an overlapping coding region featuring two tandem UGA codons, immediately followed by an RNA region that is the inverse complement (antisense) to a region of the mRNA of the selenoprotein iodothyronine deiodinase II (DIO2). This antisense interaction was confirmed in vitro via electrophoretic gel shift assay, using cDNAs at the EBOV and DIO2 segments. The formation of a duplex between the two mRNAs could trigger the ribosomal frameshift, by mimicking the enhancing role of a pseudoknot structure, while providing access to the selenocysteine insertion sequence (SECIS) element contained in the DIO2 mRNA. This process would allow the −1 frame UGA codons to be recoded as selenocysteine, forming part of a C-terminal module in a low abundance truncated isoform of the viral polymerase, potentially functioning in a redox role. Remarkably, 90 bases downstream of the −1 FS site, an active +1 FS site can be demonstrated, which, via a return to the zero frame, would enable the attachment of the entire C-terminal of the polymerase protein. Using a construct with upstream and downstream reporter genes, spanning a wildtype or mutated viral insert, we show significant +1 ribosomal frameshifting at this site. Acting singly or together, frameshifting at these sites (both of which are highly conserved in EBOV strains) could enable the expression of several modified isoforms of the polymerase. The 3D modeling of the predicted EBOV polymerase FS variants using the AI tool, AlphaFold, reveals a peroxiredoxin-like active site with arginine and threonine residues adjacent to a putative UGA-encoded selenocysteine, located on the back of the polymerase “hand”. This module could serve to protect the viral RNA from peroxidative damage.

## 1. Introduction

Over the past few decades, the Ebola virus has reemerged across the world in several epidemics, generating severe symptoms with high mortality rates and warranting extensive study [1,2]. The Zaire ebolavirus (EBOV) is an RNA virus, a class associated with emerging virus outbreaks and epidemics due to their small RNA genomes, allowing for fast, error-prone replication, and, thus, a high rate of mutation. This facilitates efficient adaptation for transfer between species, and for combating host immune systems [3]. This non-segmented, negative-strand RNA virus contains a genome of 7 essential proteins flanked by 3′ leader and 5′ trailer regions [4,5].

**Figure 1:**
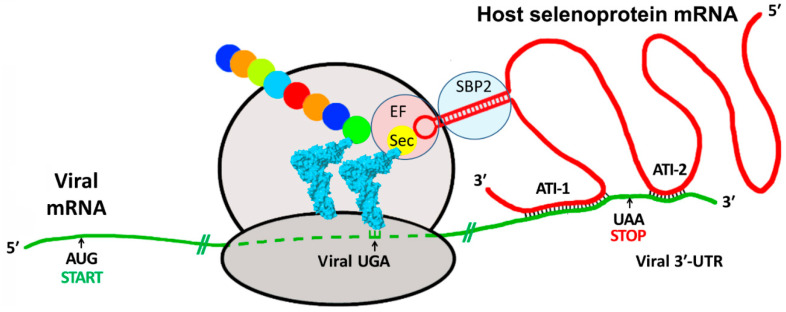
Schematic of the proposed mechanism for viral mRNA capturing the SECIS element of a host selenoprotein mRNA. As proposed by Taylor et al. [6], viral mRNA (green) is bound to the ribosome and is tethered downstream to a host selenoprotein mRNA (red) by one or more antisense tethering interactions (ATIs), which can potentially be in either a protein coding region or in a 3′ untranslated region. The captured host mRNA SECIS element (red hairpin) can then enable the viral mRNA to recode its UGA stop codon as selenocysteine (Sec), adding it to the growing peptide chain (colored circles) instead of terminating translation. Abbreviations: EF = elongation factor; SBP2 = SECIS binding protein 2.

Our previous work with EBOV focused on the 3′ end of the nucleoprotein (NP) gene, which shares some unusual structural features with the HIV-1 *nef* gene [6]. Both terminate in UGA stop codons that are remarkably well conserved within the most virulent subtypes (the main group M of HIV-1 subtypes, and the Zaire subtype of Ebola), and, near the 3′-UGA stop codon, both feature mRNA regions with antisense complementarity to human mRNA encoding isoforms of the selenoprotein thioredoxin reductase. As shown schematically in Figure 1, these antisense interactions were suggested to serve the purpose of tethering the host selenoprotein mRNAs to capture their SECIS elements, allowing reprogramming of the nearby viral UGA codon as selenocysteine [6,7]. For both these sites, translational readthrough of the 3′ UGA stop codons of HIV-1 nef and EBOV NP expression constructs has been validated in vitro using green fluorescent protein (GFP) reporter gene assays [7,8].

Using computational methods to identify potential ribosomal FS sites in RNA viruses, we have also found that such sites are, in some cases, associated with UGA codons in the −1 reading frame, that may code for selenoprotein variants of the proteins encoded in the zero frame [9,10,11]. The concept of SECIS capture by antisense tethering of a host selenoprotein mRNA [6] provides a long-sought explanation for how such viral selenoprotein variants could be synthesized, despite a lack of SECIS elements in RNA viruses. In this work, we present evidence that a potential viral selenoprotein may be translated from a novel ribosomal FS site in the EBOV L gene, at the 5′ end of the genome, which encodes the viral RNA-dependent RNA polymerase (RdRp) as a single mRNA transcript [12].

### 1.1. Mechanisms of Ribosomal Frameshifting

During translation, the ribosome can move from one frame to another in a frameshift, which can happen accidentally or be “programmed” for the deliberate purpose of encoding a protein variant. This event occurs at specific sites in the mRNA, characterized as a “slippery” or “shifty” sequence, which in the case of −1 frameshifting, is ideally a heptamer with the motif X XXY YYZ in the 0 frame, where X is any base, Y is A or U, and Z is not G [13]. This pattern allows the mRNA strand that is initially bound to its complementary A and P site aminoacyl-tRNAs to slip backwards into the −1 frame and bond with the adjacent codons, while maintaining cognate or near-cognate codon–anticodon pairing. This is in part due to the wild-card character of the third “wobble” position in the codon. Almost all the amino acids have synonymous codons that can vary at that position, e.g., some with any purine (A or G) being acceptable, others allowing either C or U, and about a third of the amino acids allowing any of the four bases in the wobble position. This design, and the fact that GU base pairs are very common in RNA structures, makes the acceptance of frameshifted base pairings more feasible, if the first two bases of each codon are the same before and after the shift. However, a slippery sequence alone only provides a low efficiency (1–2%) basal signal for frameshifting, which additionally requires certain RNA structures to significantly enhance FS efficiency [14,15].

**Figure 2:**
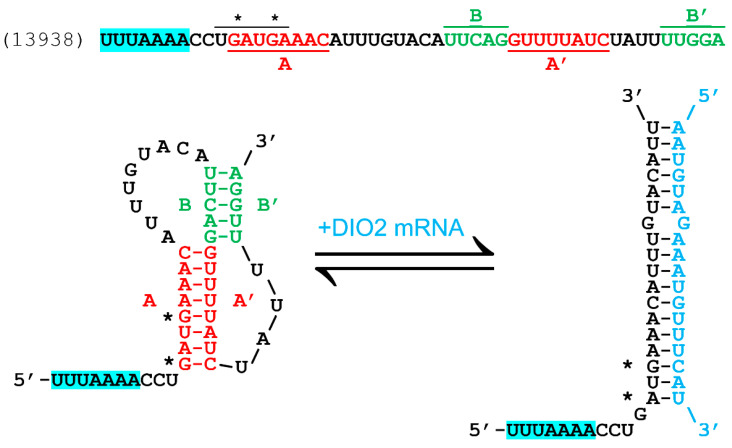
Previously predicted FS site in the L gene of Ebola Zaire. The slippery sequence heptamer (UUUAAAA, in blue highlight) is a perfect match to the X XXY YYZ pattern that is observed in most −1 FS sites. Downstream, after a few bases, is a region that can form a pseudoknot, a structure formed from two interlocking stem–loops. The first stem is formed by the pairing of sequences A and A′ (red) and the second stem is formed from the B and B’ sequences (green). These can form the secondary structure at bottom left. In the alternative base pairing shown at bottom right, the same region of the L gene mRNA is also an antisense match to a region of the human deiodinase II (DIO2) mRNA (blue). The host mRNA in this pair includes an SECIS element that could facilitate recoding (as selenocysteine) of the tandem UGA codons (*) in the overlapping region of the L gene accessed by the frameshift. Thus, DIO2 mRNA binding could be a trigger for a FS event enabling the expression of a viral selenoprotein module [6,20].

The probability that slippage can occur increases when the ribosome is stalled during translation, giving the codon:anticodon pairs time to separate and then potentially re-pair with a new triplet one base forward or back. There are several ways that this ribosomal stalling can occur, which may be as simple as when a particular amino acid is at sufficiently low cytosolic concentration, causing a delay (called “hungry codon” frameshifting). For this mechanism, the second zero frame codon is for an amino acid subject to scarcity [16]. Most commonly, especially for −1 frameshifting, ribosomal pausing is induced by a downstream pseudoknot structure [15,17,18]. The pseudoknot is an interlocking stem–loop structure that can form from the mRNA that is being translated, typically separated from the slippery sequence by a 5–8 nucleotide spacer region [19]. The pseudoknot typically forms from 2 interlocking stem loops, where the first loop forms part of the second stem [18], as shown for the site of interest in the EBOV L gene in Figure 2.

The efficiency of frameshifting events is usually low—sometimes as low as 0.5%—but can be as efficient as 80%, though in most cases the resulting protein is in much lower abundance as compared to the relative zero frame product [21,22,23]. This mechanism allows a virus to encode for multiple proteins via multiple overlapping reading frames within a single genomic sequence. For example, the HIV gag–pol fusion protein, much like the murine leukemia virus, is expressed at a ratio of 1:20 relative to the gag protein. The pol gene is out of frame to the gag gene and is expressed only 5% of the time when an FS occurs [14]. This is a useful mechanism used by many other viruses to increase protein coding density within a very small genome [14,24,25].

Potential −1 FS sites can be identified using an algorithm that first searches for the consensus −1 FS “XXXYYYZ” pattern and then for a nearby downstream region with internal complementarity capable of forming pseudoknot structures, e.g., as implemented in the KnotInframe program [26]. Using the University of Bielefeld Bioinformatics Server (https://bibiserv.cebitec.uni-bielefeld.de/knotinframe; accessed 1 September 2014), the potential FS sequence shown in Figure 2 was previously identified in the EBOV L-gene using that method [20]; that site prediction as output by KnotInframe is reproduced as Figure S1. Immediately downstream of the FS heptamer in the −1 reading frame of this site are two tandem UGA codons (* in Figure 2), so that an FS at this site would give a truncated polymerase enzyme, via their action as stop codons. However, if these could be recoded as selenocysteine, cysteine or another amino acid, an additional FS product extended by 28 residues could be formed, ending at a proline codon and a UAA stop (CCUUAA) in the −1 frame, relative to the polymerase protein. 

### 1.2. Antisense Tethering Interactions

Viruses use nucleotide base-pairing interactions between their own mRNAs and host mRNA to manipulate the expression of the host proteins as well as affect viral protein expression and genome replication [27,28,29]. This interaction relies on the inverse complementarity of virus/host RNA:RNA sequences, and potential interactions can be found computationally within a set of host and pathogen genomes. Most of the focus of research in this area has been on microRNAs, which generally serve to downregulate targeted mRNAs, and can be host or virus encoded [27,29]. However, RNA:RNA antisense interactions can serve other important functions, including the enhancement of ribosomal frameshifting, as has been demonstrated in several systems [30,31]. One such example that is particularly relevant here is the case of an essential FS in SARS coronavirus, where mutation of residues involved in a kissing-loop dimerization of the viral mRNA reduces FS efficiency by 62% [32].

Our group has proposed that virus–host RNA antisense interactions could serve the following purpose: the capture of functional host RNA structural elements to serve a viral agenda, consistent with the universal viral strategy of hijacking host components for virus production [6]. As discussed above, our prototypical example of this mechanism (shown schematically in Figure 1) involved sites in the mRNAs for HIV *nef* and the EBOV nucleoprotein, with the potential to capture host SECIS elements via antisense tethering interactions (ATI), that were found to be adjacent to the conserved 3′-UGA stop codons for the respective viral genes [6]. A focus of the current study is another potential ATI that was identified involving a region of the Ebola L gene and the mRNA for the human selenoprotein deiodinase II (DIO2) [20], which proved to be adjacent to the −1 FS site, as shown in Figure 2.

### 1.3. Internal Protein Domain Swapping Encoded by Dual Opposed Programmed Frameshifts 

Programmed ribosomal frameshifting allows the assembly of alternate protein modules, similar to alternative RNA splicing or “exon shuffling”, with the alternative domains encoded in overlapping reading frames of a single RNA sequence, rather than in separate regions of a pre-mRNA. Frameshifting is less versatile; in known examples, it only serves to substitute a protein domain at the C-terminal of a protein, downstream of the FS site. Thus, frameshifting only permits possibilities like AB plus AC, whereas alternative RNA splicing also allows for possibilities like ABC plus AB or AC, where A, B, and C are different protein domains.

However, this limitation of frameshifting only applies in the case of a single frameshift. To our knowledge, the possibility of dual opposed frameshifts, with the second shift returning to the original reading frame after inserting an alternative module encoded in the overlapping frame, has never been considered. This would permit *internal* protein domain swapping similar to what can be accomplished by alternative RNA splicing. Structure–function considerations led us to ask whether this swapping could be occurring in the EBOV L gene, where, 90 bases downstream of the −1 FS site shown in Figure 1, we identified a putative +1 FS signal similar to known sites of forward P-site slippage on proline codons when followed by a stop codon, e.g., CCCUGA [33]. A return to the zero frame would enable the attachment of the entire C-terminal of the polymerase protein, forming an essentially complete L gene multienzyme complex with a small region of the polymerase palm domain modified, mostly on the back of the polymerase “hand”.

The structural and functional implications of these findings will be analyzed and supported with computational and experimental evidence, including evidence for conservation of the sequence features involved, and 3D modeling of the predicted EBOV polymerase FS variants using the AI tool, AlphaFold2 [34].

## 2. Materials and Methods

### 2.1. Sequence Analysis and Assessment of Antisense Matches

Potential antisense interactions between host and virus RNAs were initially identified using BLAST searches (http://blast.ncbi.nlm.nih.gov/Blast.cgi, accessed on 1 May 2024). The EBOV genome was used as a query against human targets using the nucleotide–nucleotide option (blastn), starting with default parameters against the Reference RNA Sequence Database (refseq.rna), restricted to the target of human RNA (*Homo sapiens* taxid: 9606) or other taxa of interest. Because RNA:RNA interactions are more complex than the simple DNA-based complementarity [35], potential antisense matches identified using BLAST were further assessed using the web-based tool RNAHybrid 2.2 (https://bibiserv.cebitec.uni-bielefeld.de/rnahybrid, accessed on 1 May 2024), which uses parameters optimized for RNA folding to find the most stable and favorable regions of interaction between two RNA sequences, e.g., by allowing for GU base pairs and ranking alternative stem–loops that can form in dsRNA. To further assess potential RNA secondary structures associated with possible frameshift sites, RNA folding was performed using RNAstructure version 6.5 with default parameters (https://rna.urmc.rochester.edu/RNAstructure.html, accessed on 10 April 2024).

BLAST was also used to assess the degree of conservation of the FS heptamer and specific codons in the overlapping reading frame, e.g., by using the EBOV sequences of interest as probes vs. the complete set of full-length EVOV genomes in the NCBI nucleotide collection, using default BLASTN parameters, specifying ZEBOV (taxid:186538) as the organism, and “complete genome” as the Entrez keyword.

### 2.2. Gel Mobility Shift Assay

Based on the computational results, the oligos used in the gel shift assay were bases 13937–13967 from the Ebola virus (NCBI NC_002549), named oligo E, and bases 31511–31541 from human DIO2 (NCBI NC_000014.9), named oligo D. A randomly shuffled version of the EBOV sequence was generated and named oligo R. Oligos were ordered from Integrated DNA Technologies, Inc., Coralville, IA, USA.

The lyophilized DNA was dissolved in PBS to create 1 μM stock solutions, which were stored at 4 °C. One microliter of each sample (D, E, or R) was further diluted in PBS to a total volume of 10 μL (at 100 nM). Oligos, either singly or in pairs of equal volumes of D + E, D + R, E + R, or D + E + sheared herring sperm DNA (Sh), were incubated at room temperature for 1 h. The tubes were heated at 66 °C for 15 min and then cooled at room temperature for 40 min. Along with 2 μL of loading dye, the DNA was placed into the lanes, using 10 μL of either the single strands or pair mixtures, giving about 1 pmol of total DNA per well. The 5% TBE agarose gel, with ethidium bromide, was run at 65 v for 4 h while the setup was chilled in ice water. The gel was then analyzed and imaged with a BIO-RAD Gel Doc™ XR+ Molecular Imager (Hercules, CA, USA).

### 2.3. Frameshift Construct Design

**Figure 3:**
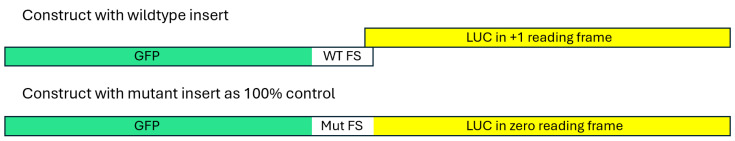
Schematic of the protein expression cassettes in the dual reporter FS assay construct. Translation begins with a green fluorescent protein (GFP) reporter gene (green) upstream of the viral L gene insert containing the putative FS site; downstream of the viral insert is the luciferase (LUC) reporter gene (yellow). With appropriately designed inserts and controls, this approach enables assessment of the ratio of the proteins translated in the zero vs. either the −1 or +1 reading frames. Shown here are the designs used to assay for a predicted +1 FS site.

The standard approach for cell-based in vitro FS assays involves a construct with upstream and downstream reporter genes flanking a central insert containing the putative FS sequence [36]. Whenever protein synthesis is initiated, the upstream gene is expressed, providing a standard for normalizing the amount of downstream reporter gene expression, independent of cell numbers and transfection efficiency. Appropriately designed mutant inserts provide controls for 100% readthrough (equal molar ratio of both proteins), so that the efficiency of frameshifting in the wild type insert can be estimated (Figure 3).

For reasons discussed in the results section, due to the UGA stop codons in the −1 frame, this approach could not be applied to the putative −1 FS site shown in Figure 2. However, we were able to use this method to validate a predicted +1 FS site about 90 bases downstream of the −1 site (Section 3.3).

The viral inserts for the +1 FS assay constructs were as follows:

Wild Type, which only produces LUC if a +1 FS occurs at the slippery sequence (yellow) is described as follows:

5′AAGCTTAACATTTGTACATTCAGGTTTTATCTATTTTGGAAAAAAACAATATTTGAATGGGGTCCAATTGCCTCAGTCCCTTAAAACGGCTACAAGAATGGCACCATTGTCTGATGCAATTTTTGATGATCTTCAAGGGACCCTGGCTAGTATAGGCACTGCTTTTGAGCGATCCATCGAATTC3′

The FS knockout mutant, in which deletion of one T from the slippery sequence heptamer (yellow) brings Luc in-frame with GFP as the 100% readthrough control is described as follows:

5′AAGCTTAACATTTGTACATTCAGGTTTTATCTATTTTGGAAAAAAACAATATTTGAATGGGGTCCAATTGCCTCAGTCCCTAAAACGGCTACAAGAATGGCACCATTGTCTGATGCAATTTTTGATGATCTTCAAGGGACCCTGGCTAGTATAGGCACTGCTTTTGAGCGATCCATCGAATTC3′

The insert spans the EBOV sequence (13953-14124 in NCBI NC_002549) extending in both directions beyond the putative +1 FS site (CCTTAA). This sequence is to include as much local RNA structure in the insert as possible, and to allow some space between the restriction site and the potential pseudoknot formation (see Results Section 3.3). A HindIII restriction site was added (in blue) to the 5′ end, and an EcoRI restriction site (purple) was inserted at the 3′ end. Underlined (in yellow) is the slippery sequence, CCTTAA, in the wildtype insert. As a positive control for 100% readthrough without frameshifting, the second T nucleotide (14029) in the slippery sequence was deleted in the mutant construct. Where the wildtype contained a shifty proline-stop codon sequence at this position, the removal of the T in the mutant control plasmid eliminates the UAA stop codon that causes the ribosome to pause during translation, and instead creates a proline–lysine in that frame, returning the reading frame to the L gene protein sequence. Upstream of the viral inserts, in the zero frame of both constructs, GFP was used as the standard for the initiation of protein synthesis. The LUC was added downstream of the viral inserts, in the zero frame for the mutant and in the +1 frame for the wildtype. If frameshifting occurs in the wildtype, then luminescence will be expressed because of a shifting to the new reading frame that contains the LUC reporter gene. A control for the baseline of luminescence relative to GFP was made that only contains the GFP sequence, without the downstream luciferase. These full inserts were then ligated into a pAcGFP1-N3 plasmid containing a T7 promoter and an ampicillin-resistance gene. The procedure results in a circular plasmid when fully assembled. The viral inserts were ordered from IDT (Integrated DNA Technologies, Inc., Coralville, IA, USA). The constructs were assembled by Custom DNA Constructs plasmid cloning company (Islandia, NY, USA).

### 2.4. Plasmid Propagation

Full constructs of both wildtype and mutant were propagated using JM109 bacterial cells (Promega, Madison, WI, USA, Catalog number L2005). and purified using a PureYield^TM^ Plasmid Miniprep system by Promega (Catalog number A1222). A total of 50 μL JM109s were thawed on ice for 30 min then mixed with 1 μL of plasmid DNA at 250 ng/μL for an additional 30 min. Cells were then heat shocked by placing them at 42 °C for 45 s then on ice again for 2 min. The bacteria were then added to 1 mL of Super Optimal broth with Catabolite repression media and incubated at 37 °C for 45 min while shaken. The outgrowth solution was then added onto an LB agar plate by T-stroking and incubated for 24 h at 37 °C. Isolated colonies were picked and grown in Luria Broth with 50 ug/mL ampicillin overnight. The manufacturer’s protocol was performed, eluting with 30 μL of heated nuclease-free water. Plasmid concentrations were measured with a DS-11 spectrophotometer (DeNovix, Wilmington, DE, USA) and stored at −4 °C. 

### 2.5. Transfection Protocol

Cells were seeded into four groups (Untransfected, Wildtype, Mutant, and Zero-Luciferase) of six wells each in 96 well plates at 100,000 cells per well, with 100 μL of media, and incubated for 24 h at 37 °C at 5% CO_2_. The 1 μg of DNA for each of the three plasmids was placed into three separate tubes containing 100 μL of 25 ng/μL PEI and filled up to a total of 120 μL of Opti-MEM (Gibco catalog number 31985070). An additional 10 μL of Opti-MEM was added to each of these three tubes. A fourth tube for the untransfected group was made with 100 μL of the same PEI with 30 μL of Opti-MEM. After mixing, the tubes were incubated at room temperature for 30 min. The 20 μL of the lipid–DNA complexes was then added to the appropriate wells, after which the cells were incubated again for 48 h. A media change was performed 24 h after transfection.

### 2.6. Reporter Gene Assay

After transfection and incubation, cells were lysed with 40 μL Reporter Lysis Buffer (Promega, catalog number E4030) and incubated at 37° C at 5% CO_2_ for 15 min. Fluorescence was measured first using a Flexstation II multimode plate reader (Molecular Devices, San Jose, CA, USA). The following settings were used: Fluorescence mode; Well Scan; Pattern: Fill; Density: 3; Spacing: 1.13 mm; Total Points: 9; Top read; Wavelengths: Ex. 485 nm, Em. 538 nm, auto cut-off 530 nm; Automix: Off; Calibrate: On; PMT: Auto; Reads/Well: 30; RFU Min: 0; RFU Max: 20,000 Assay Plate Type: 96 Well blk/clrbtm; Wells to Read: (selected wells only); Column Wavelength Priority: Column Priority; Autoread: Off.

After the GFP was read, luminescence was read with the plate reader after adding reagents from the ONE-Glo™ Luciferase Assay System (Promega, catalog number E6110) prepared using manufacturer’s instructions. A total of 20 μL of 1× substrate was added to each well and incubated at room temperature for 15 min. After luminescence was selected, the settings were the same as for fluorescence.

### 2.7. Modeling of EBOV L Gene Protein Frameshift Isoforms Using AlphaFold2

The protein sequence and residue numbering scheme used for modeling were from the EBOV RdRp NCBI reference sequence NP_066251, derived from the 1976 EBOV genomic reference sequence NC_002549. Isoforms C and D were modeled separately by submitting the respective predicted protein sequences to AlphaFold2 [34] using the following Colabfold notebook in Google Colab [37]: https://colab.research.google.com/github/sokrypton/ColabFold/blob/main/AlphaFold2.ipynb, accessed on 1 May 2024.

The tandem UGA codons in the FS encoded module were translated as cysteine in these sequences, as selenocysteine is not recognized by the program. The full protein sequences submitted for the two FS isoforms are given in Table S1. The resulting structures were refined using the AMBER force field “relax” option in AlphaFold. Molecular graphics were generated using Maestro 10.7 (Schrodinger, Inc., New York, NY, USA).

## 3. Results and Discussion

### 3.1. Identification of EBOV L Gene -1 Frameshift Site and Antisense Complementarity to DIO2 

The previous analysis of the FS site, using KnotInFrame ([20]; results are included here in Figure S1), identified the following two important features in addition to the X XXY YYZ heptamer and potential pseudoknot: two −1 frame UGA codons and antisense complementarity to an mRNA region from a human selenoprotein, iodothyronine deiodinase II (DIO2), as shown in Figure 2. It is unlikely to be a coincidence that both these features are found so close together, because the two UGAs could be recoded as selenocysteine, but would need an SECIS element, not present in the viral genome, to enable its recoding. The DIO2 mRNA, which is complementary to the precise region of the L gene where the two UGAs are located, contains an SECIS element that could be recruited for this purpose via antisense tethering [6]. However, since both UGAs are in the −1 frame, an FS needs to occur, which is possible due to the presence of the ideal slippery sequence heptamer UUUAAAA just a few bases upstream (Figure 2). The antisense tethering interaction could recruit the SECIS element needed for recoding the UGAs, while simultaneously providing the necessary secondary structure (as shown in bottom right of Figure 2) to pause the ribosome long enough for an FS to occur, acting similarly to a pseudoknot, i.e., as an FS enhancer. This antisense-enhanced FS mechanism has been previously demonstrated with synthetic constructs [30,31] and in SARS-CoV-2 [32].

**Figure 4:**
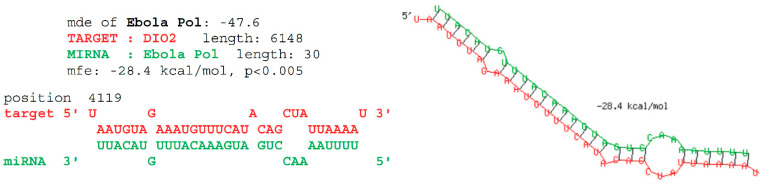
Predicted antisense interaction between regions of human DIO2 and Ebov L gene mRNAs. The interaction was initially identified using BLAST, then assessed further using RNAHybrid 2.2 [20]. The predicted interaction (*p* < 0.005) is shown as both a sequence alignment and in secondary structure format; the binding free energy was calculated as −28.4 kcal/mol (*p* < 0.005). For comparison, the Ebola Pol MDE of −47.6 kcal/mol represents the lowest (best) possible binding energy for that fragment, calculated by binding the Ebola region shown to its Watson–Crick inverse complement.

The predicted antisense interaction between the L gene of the Zaire Ebola virus (MN416402.1) and DIO2 was also identified previously [20] and is shown in detail in Figure 4.

### 3.2. In Vitro Assessment of the Predicted Antisense Interaction

**Figure 5:**
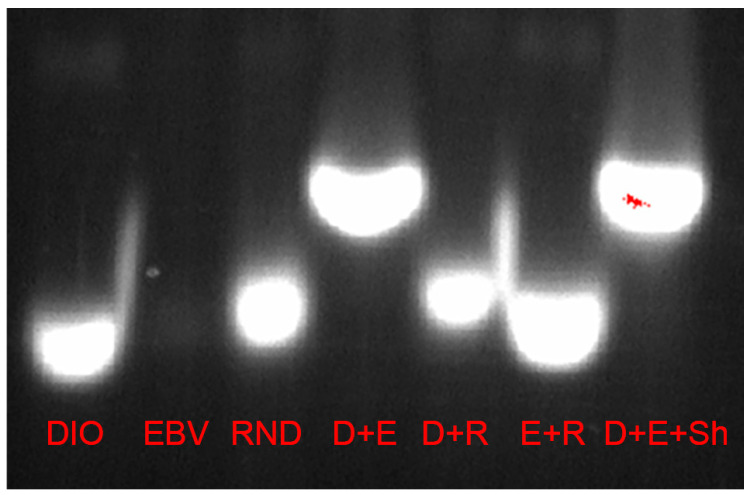
Gel mobility shift assay validating the antisense tethering interaction between regions of human iodothyronine deiodinase II cDNA and Ebola polymerase (L) cDNA. The red “hot pixels” are an artifact of the intense light signal at those points.

The potential antisense interaction shown in Figure 4 was experimentally validated via a gel mobility shift assay, using DNA oligos as a model for the RNA:RNA interaction (Figure 5). The two DNA oligos corresponding exactly to the fragments shown in Figure 4 were found to be complementary in solution, forming duplex DNA, unlike a randomly shuffled control of identical base composition, which remained unpaired. This result is consistent with the hypothesis that this is a functional antisense interaction in EBOV-infected cells that express DIO2.

The labels in each lane represent different single-stranded DNA oligonucleotides, where (DIO or D) is human deiodinase II, (EBV or E) is the Ebola L gene, (RND or R) is a randomized sequence of DIO, and the right lane includes sheared herring sperm DNA (Sh), a complex of DNA used to represent the cellular environment of competing oligonucleotides. Paired lanes contained both corresponding oligos. All single sequence lanes and both the E + R and D + R lanes migrate as single strands, whereas D + E migrates more slowly as a duplex. This indicates that the antisense interaction occurs between the DIO and EBV oligos, as they are migrating as the sum of the 2 single-strand weights instead of moving together as single strands as seen in the D+R and E+R lanes. The 2 oligos in the shear lane are still able to tether despite the abundance of alternative DNA, indicating the interaction is most favored between the two. Due to its lack of internal secondary structure (confirmed by RNA folding, see legend to Figure S3), EBV alone is labelled very faintly by ethidium bromide, an intercalating agent, but EBV combined with equimolar DIO causes the single-strand DIO band to disappear (D+E lanes), proving the presence of EBV by complete conversion of DIO to the hybridized duplex form.

### 3.3. Frameshift Site Validation

The standard method for assessing potential frameshifts involves dual reporter genes spanning a putative FS insert, as in the design used here (Figure 3). However, this approach could not be utilized in the case of the −1 FS shown in Figure 2, because the FS immediately leads to stop codons in the −1 frame that would prevent expression of the downstream LUC reporter gene. In the past, we have been able to show frameshifting in special cases of similar situations because the UGA codon in the overlapping reading frame did not impinge on the predicted secondary structures of the pseudoknot, so they could be mutated to sense codons without affecting the FS (e.g., see [9]). In the case of the EBOV L gene, the tandem UGA codons map to helical regions of both the pseudoknot and the alternative antisense pairing (Figure 2), which would be disrupted by their mutation to sense codons. Thus, we are not able to directly validate frameshifting at that site via this standard method. However, it is known that an ideal −1 FS heptamer alone, as we have here (UUUAAAA), provides a basal level of FS activity of 1–2% [15], which would then be enhanced by downstream RNA structures. Furthermore, we have experimental support for the antisense interaction that could be an enhancer of the FS (Section 3.2).

**Figure 6:**
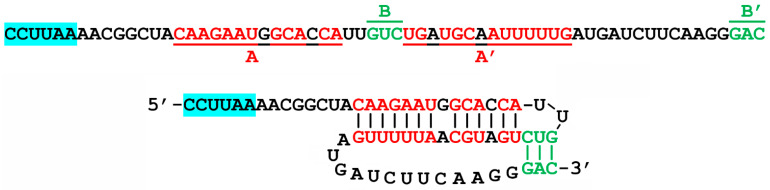
Potential +1 FS site in EBOV L gene at the 3′ end of overlapping coding region in the −1 frame. The primary FS signal is the shifty proline codon followed by the stop codon, CCUUAA. The RNA stem A:A’ may be an additional enhancer of frameshifting and is predicted by RNA folding, along with a second stem that has the potential to form the B:B’ stem in the pseudoknotted structure shown here, as a kissing loop between stem A:A’ and a downstream stem (see Figure S2). The entire RNA region shown in Figure S2 was included in the L gene insert used in the FS assay to validate +1 frameshifting at this site.

However, we were able to obtain experimental evidence in support of frameshifting at a putative +1 FS site that is 90 bases downstream, which can only come into play if translation has already shifted to the −1 reading frame. Thus, this finding provides indirect evidence that the first (−1) FS must occur, since otherwise the structural features that enable functional +1 FS activity at the second site (Figure 6 and Figure S2) could not be selected and conserved during evolution.

**Figure 7:**
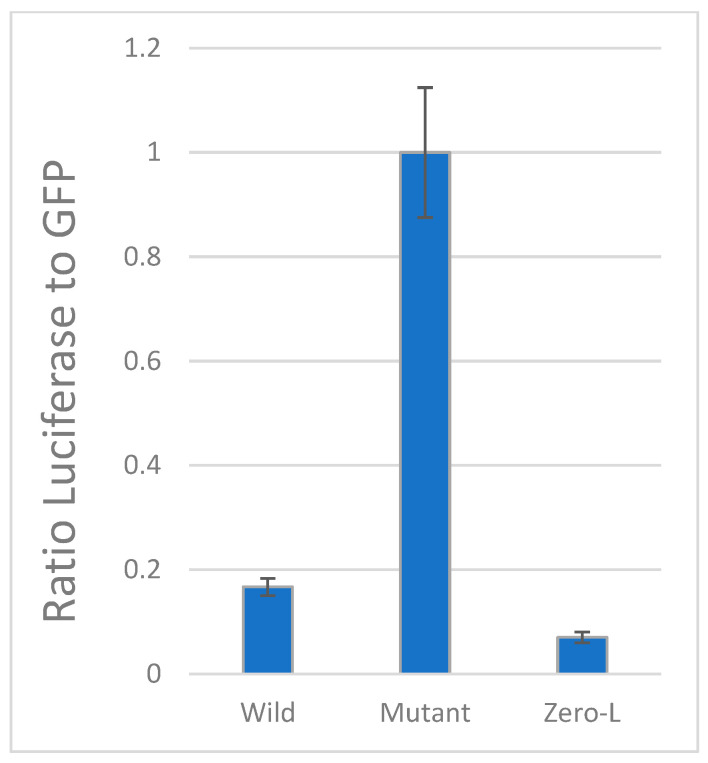
Evidence for an active +1 frameshift site in the EBOV L gene. The bars correspond to the ratio of luciferase (LUC) to GFP expression in cells transfected with wildtype or mutant dual reporter assay constructs, or not expressing LUC as a baseline control (Zero-L). Data are the mean of 3 independent experiments ± SEM, each of which was conducted with 5 or 6 replicates. The ratio of LUC/GFP signals was normalized to Mutant = 1 in each separate experiment, as that represents 100% readthrough from GFP into the LUC regions. In the mutant control construct, this results in a significantly higher LUC production over the wildtype sequence (*p* = 0.003 by unpaired *t*-test). The wildtype construct still produced significant LUC relative to the Zero-L control (*p* = 0.005), confirming that +1 ribosomal frameshifting is induced by this L gene fragment, at about 10% efficiency.

The predicted +1 site from Figure 6 was found to be functional using the dual reporter assay, as shown in Figure 7 (average data from each experiment in Table S2). In all conditions, GFP is produced upon initiation of translation, and LUC is measured relative to GFP production. With the wild type insert, LUC is in the +1 reading frame and is only expressed when the FS event occurs. In the mutant 100% control construct, due to deletion of a U base in the CCUUAA FS hexamer, LUC is translated in equimolar amounts to GFP. Their respective activity relative to background Zero–LUC control suggests that there is a significant level of +1 frameshifting at this site, at about 10% efficiency (*p* = 0.005).

It is well established that proline codons are exceptionally prone to frameshifting, particularly when followed by a pause-inducing stop codon, e.g., CCCUGA [33]. For that CCC proline codon, after a +1 FS to CCU, there would only be a mismatch in the wobble position, which considering the degeneracy of the CCN proline codon family, readily facilitates frameshifting. In the current case of CCUUAA, a tRNA decoding CCU would have to pair with CUU after slippage. For proline tRNAs that optimally decode CCU, with an AGG anticodon (the human tRNA^Pro^ gene *TRP-AGG1-1*), on CUU there would still be only a single mismatch, in the center codon position, and it would be a GU base pair, which are very common in RNA structures. The P-site slippage on the CCUU tetramer may be enhanced by the pausing effect of both the UAA stop codon and downstream RNA structures that we have identified (Figure 6 and Figure S2).

### 3.4. Predicted L Protein Isoforms Resulting from Possible Frameshifting and Stop Codon Readthrough

**Figure 8:**
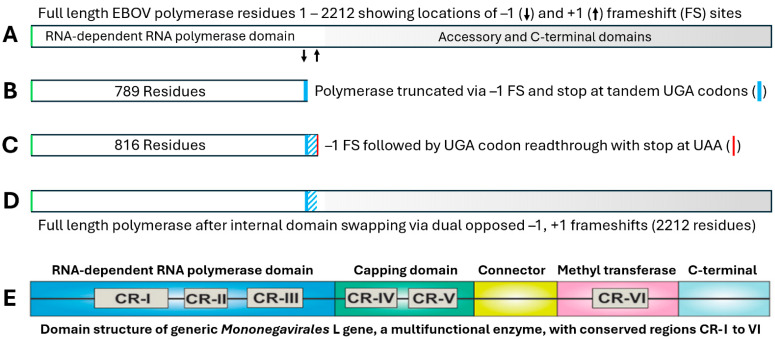
Predicted L protein isoforms resulting from single or dual opposed frameshifts with and without UGA stop codon readthrough. See text for details. Despite substantial truncation, isoforms B and C have the potential to possess functional RNA polymerase activity. Calculated molecular masses of the truncated isoforms B and C are 90 and 94 kDa, respectively. Isoforms C and D include a small 28-residue module with potential redox-associated function (hatched blue region), that is encoded in the −1 reading frame of the L gene. Panel E is modified from Figure 2A of Liang [38], CC by 4.0.

Our findings presented above imply the existence of several previously undetected isoforms of the EBOV L gene product, which can be better understood by a structural analysis in relation to the known domain structure of the *Mononegavirales* L gene, which is highly conserved across a number of viral species [38]. The L gene encodes a multifunctional enzyme complex, shown schematically in Figure 8E. Figure 8 shows the following: (A), the wild type polyprotein; (B), a truncated 90 kDa isoform resulting from the −1 FS from Figure 2, terminating at the first UGA codon in the −1 frame; (C), a truncated 94 kDa isoform resulting from the −1 FS followed by recoding of the tandem UGA codons in the −1 frame, terminating at a proline after 28 residues encoded in the −1 frame; and (D), a full-length-variant L protein isoform in which synthesis of isoform C is followed by a +1 FS at the terminal proline/stop codon hexamer from Figure 6, returning to the zero frame of the L protein. This creates a full-length L protein isoform in which a small internal domain of 28 residues has been swapped for one of identical size encoded in the overlapping −1 reading frame. Because predicted isoforms B and C are truncated close to the end of the core RNA dependent RNA polymerase (RdRp) domain, it is possible that this first (−1) FS site is situated to produce a core RdRp module that is not attached to the downstream enzymes, which serve functions of RNA capping and methylation. Although the capping domain is considered part of the polymerase “thumb”, it has been noted that its “priming loop” and supporting structures have to swing out from the active site during the elongation phase to enhance highly processive RNA synthesis [38]. Hence, RdRp isoforms omitting the cap and downstream domains could play a role in more efficient transcription or replication of the viral genome. Significantly, the RNA capping and methylation functions only apply to the transcribed subgenomic mRNAs, whereas the antigenome replication intermediate, the genomic RNA, and small leader RNAs are uncapped [38]. Hence, if they are able to function as a minimal polymerase, FS isoform B or C could serve a specialized function in the synthesis of those uncapped viral RNAs.

Using conventional techniques (other than mass spectrometry) such as Western blot, isoform D would be virtually impossible to differentiate from the conventional isoform A, as their molecular masses would be too close to resolve on a 1D gel. However, isoforms B and C could have been detected in some published work on Ebola. Unfortunately, as such work requires a biosafety level 4 lab, the number of suitable papers potentially containing such results is limited. Our search turned up only a single paper with a Western blot of the L protein, expressed along with VP30 and VP35 [39]. The key panel in question, Figure 1C of Tchesnokov et al. [39], has a very strong band at 90 kDa, which the authors attribute to heat shock protein 90 (HSP90), which is known to associate with viral polymerase gene products. However, it is by far the widest and darkest band on the gel, and could easily result from a combination of HSP90 plus the 91 and 94 kDa RdRp isoforms B and C predicted by our results. Hopefully, our findings will stimulate further efforts to identify the actual products of EBOV L gene expression in infected cells or tissues.

### 3.5. Assessment of Structural Feasibility and Functional Implications of the Predicted Frameshift Isoforms via Protein Modeling Using AlphaFold2

**Figure 9:**
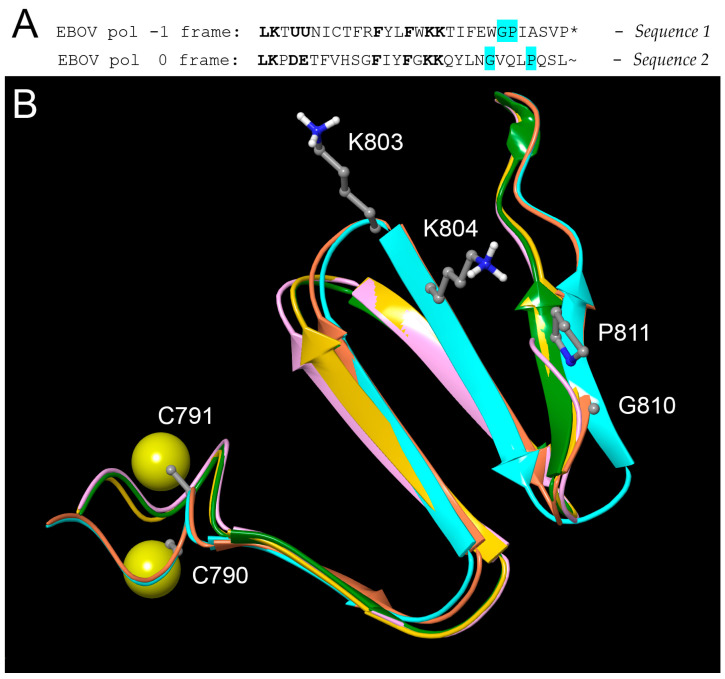
Sequence and overlapped structures of the frameshifted region from 5 AlphaFold2 models. (**A**). Protein sequence encoded in the −1 reading frame between the two predicted FS sites (*Sequence 1*), compared to the zero-frame sequence of the EBOV polymerase (*Sequence 2*). The U represents potential selenocysteine residues, encoded by the UGA codon. (**B**). Overlap of five different models generated by AlphaFold2, with the UGA codons modeled as cysteine (sulfur atoms shown in yellow). All five show a similar fold of four short beta strands. The two most highly ranked models (blue and red ribbons) are essentially identical (average Cα RMS = 0.68 Å) and differ only slightly from the other three models at the C-terminal end (right). Average Cα RMS of model 1 vs. model 5 was still only 2.4 Å. The sidechains of some important residues discussed in the text are shown.

To more accurately assess the feasibility of the predicted truncated RdRp isoforms to function as polymerase enzymes, and the possible role of the FS module containing putative selenocysteine residues, we built 3D models of predicted FS isoforms C and D using AlphaFold2 [34]. The 3D structures that AlphaFold predicts for the 28-residue domain that is “swapped” due to the FS are very similar to the structure for the corresponding region of the zero-frame protein. This finding is not surprising if one compares the two protein sequences, which share a number of similar features, despite their coding sequences being offset by one letter in the genetic code. Translation of the −1 frame-encoded product starting at the shift point results in a short sequence ending in a proline followed by a UAA stop (*). This sequence is compared below to the L gene protein sequence encoded in the zero frame that is replaced by frameshifting (Figure 9A).

The LK at the N-terminal end of both sequences 1 and 2 corresponds to the two amino acids encoded by the −1 FS heptamer U UUA AAA (Figure 2), which has tRNAs that shift and initiate entry into the −1 reading frame. These are closely followed by two acidic residues Asp–Glu (DE) in the zero-frame product, which are replaced by the tandem UGA codons, potentially translated as selenocysteine, which is also acidic, being predominantly Se^–^ at pH 7.4. The central hydrophobic clusters preceding the lysine pair (KK) are also similar (FYLF vs FIYF). Finally, in both sequences, the C-terminal regions contain two hinge or turn-associated residues, glycine and proline (highlighted in cyan). This finding is significant because modeling supports the hypothesis that this glycine is critical for a hinge motion that lifts the downstream portion of the polymerase thumb up away from the active site cleft, enabling easier access to RNA templates, which could be important for the function of this enzyme complex (see Section 3.7).

**Figure 10:**
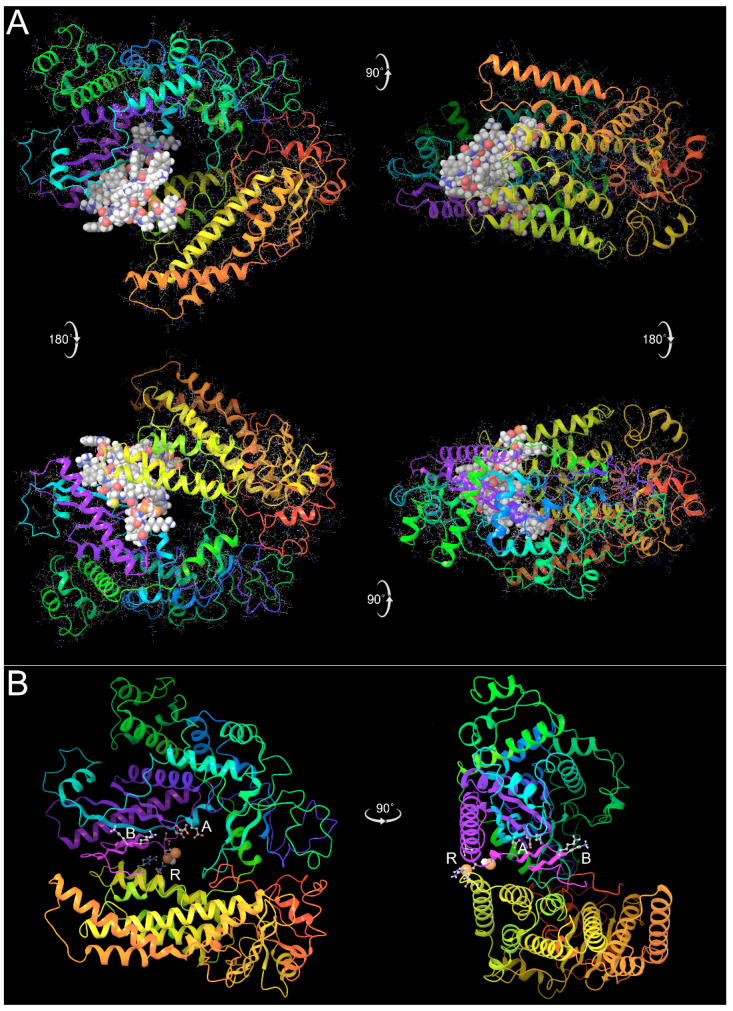
AlphaFold2 model of truncated frameshift isoform C of EBOV polymerase. (**A**). The truncated RdRp domain is rendered in ribbon (colored by position), terminating in the 28-residue module encoded in the −1 frame, shown in all-atom spacefill with CPK colors. The top left view is down into the palm of the polymerase hand, with the fingers at the top (green and cyan ribbon). Note that despite the deletion of the C-terminal thumb and downstream domains, the base of the “thumb” remains, as that region is encoded near the N-terminus of the protein (orange and yellow ribbon). The frameshifted domain is predicted by AlphaFold to occupy the same space and structure corresponding to part of polymerase Motif D and all of Motif E in the native structure, at the C-terminal end of the palm domain. (**B**). Orthogonal views of the same structure entirely in ribbon rendition, with a few key residues shown as follows: *A*, a cluster of acidic residues at the polymerase active site (Motifs A and C); *B*: a conserved pair of basic lysine residues that is identical in both the zero frame and −1 frame sequences, due to a run of seven A nucleotides in the coding sequence; and *R*, a putative redox module where two possible selenocysteine residues (in spacefill) protrude at the back of the polymerase hand.

The reliability of the AlphaFold2 model of this region, which corresponds precisely to the domain rendered in spacefill in Figure 10A, is supported by the fact that all five models generated by the program give the same fold (Figure 9B), and the average pLDDT statistic for all atoms in this 30-residue domain for the top ranked structure was 57.2. This number falls short of the score of 70 that generally assures an accurate backbone fold prediction, but combined with the consistency of the five models, supports the utility of the predicted structure as a first approximation for a totally novel protein domain.

This glycine hinge is present in both the native and dual frameshifted L gene isoforms (A and D in Figure 8). In sum, these similarities between the −1 and zero frame sequences provide an explanation for the AlphaFold results predicting that in both isoform C and D, their 3D structures are very similar in the context of the rest of the polymerase structure. The 3D structures of the Amber-relaxed Alphafold2 models of isoform C shown in Figure 10, and both the closed and open-hinged forms of isoform D discussed in Section 3.7, are included in Supplemental Material as a Schrodinger Maestro Project file.

### 3.6. Potential Redox Function of the Frameshift-Encoded Module

**Figure 11:**
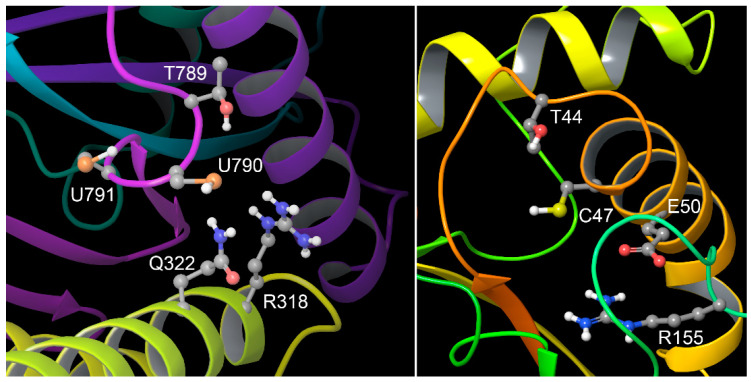
Putative −1 FS redox module compared to human peroxiredoxin 1 active site**.** Closeup of key residues in the EBOV frameshifted region (**left**) shown as R in Figure 10B, compared to that of a typical peroxiredoxin active site, from 1prx.pdb (**right**). The three universally conserved peroxiredoxin active site residues are threonine, cysteine, and arginine, with the cysteine located between the other two residues [44]. In human peroxiredoxin 1, a glutamate (E50) also participates; in EBOV, glutamine Q322 could play a similar role, and is notably also an essential active site residue of glutathione peroxidases. Note that in the EBOV redox module, R318 and Q322 are contributed to by an upstream domain that is not part of the frameshift-encoded structure, as opposed to residues 790 and 791, encoded by tandem UGA codons in the −1 frame. These are shown here as selenocysteine, but could also be decoded as cysteine under conditions of Se deficiency via a known mechanism [42]. The second selenocysteine or cysteine (residue 791) could play the role of the second, “resolving” cysteine of peroxiredoxins [44].

The Ebola L gene FS product features 2 tandem UGA codons. This motif is similar to a conserved sequence found in the following thioredoxin reductase family: -Gly-Cys-Sec-Gly [40]. This hypothetical FS protein product could serve to perform some redox related functions, which could have multiple benefits for the virus. Two tandem selenocysteines have been shown to be capable of redox activity similar to the Cys–Sec motif [41]. However, it must also be emphasized that it is possible that one or both of these UGA codons could be recoded as cysteine, using the selenocysteine insertion machinery under conditions of selenium deficiency [42]. Whether as a conventional protein or as a selenoprotein, this module could help the virus defend itself against oxidant attacks from the host immune system, as well as increase the viability of an enveloped virion by inhibiting lipid peroxidation. The most obvious function, suggested by the module’s structural association with an enzyme that both binds to and creates the viral RNA, is that of protection of the genetic material from oxidative degradation. This is a universal problem for RNA, for which cells and organisms have evolved multiple defenses and repair mechanisms, as recently reviewed [43]. Also, the possibility of a peroxidase type of function in this case seems the most likely, given the structural similarities to the essential features of a peroxiredoxin active site, as shown in Figure 11.

In addition to the general topological similarity of the FS redox module to the essential peroxiredoxin catalytic triad, with the (seleno)cysteine located between the conserved threonine and arginine residues, a more detailed geometric comparison between the two sites is given in Table S3. For this case, we examined the distances between the positions of the β-carbons of interacting residue pairs of the catalytic triad and the H-bonding Glu or Gln (Figure 10), as β-carbons are fixed relative to the backbone and not subject to change via rotation, making the assessment unbiased. As tabulated in Table S3, the deviations ∆*d* in the pairwise distances (in angstroms) between the β-carbons for the four residue pairs of interest averaged < 0.8 Å, less than 10% of the average C_β_-C_β_ distance in the peroxiredoxin structure, and about half of a C-C bond length. This finding suggests a reasonably close mimicry of the essential peroxiredoxin active site geometry.

### 3.7. A Critical Hinge Function in Polymerase Motif E Is Duplicated within the Frameshift Module

**Figure 12:**
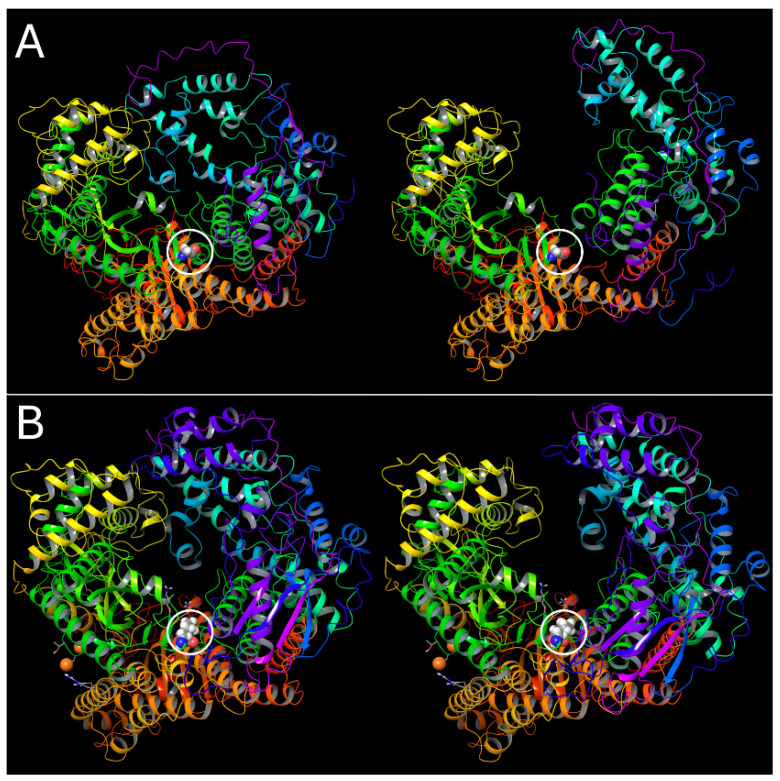
Opening of the active site cleft via a hinge motion in both the EBOV polymerase and in the swapped domain of isoform D. (**A**). *Left:* The EBOV polymerase structure from 1yer.pdb with G809 shown in spacefill, circled. *Right:* The same structure after rotation of a single backbone dihedral angle of G809 (*psi*) by 30°. (**B**). *Left:* The complete isoform D AlphaFold2 model with the GlyPro pair in the swapped domain shown in spacefill, circled. The two lysine residues that are conserved in this region in both the native and frameshifted proteins are displayed just above the circled GP pair, and the two putative selenium atoms of the frameshift-swapped redox domain are displayed as orange spheres. *Right:* The same structure after rotation of the four glycine and proline backbone dihedral angles by −6° and +34° for G810 *phi* and *psi*, and −14° and +7° for P811 *phi* and *psi*, respectively.

Following the central β hairpin of motif E, there is a glycine residue, G809, in the protein reference sequence, that is highlighted in cyan in *Sequence 2* in Section 3.5 above. Although its importance has not been noted previously, this L gene glycine is highly conserved within the family of *Mononegavirales*. As shown in Figure 12, we propose that this glycine is an essential hinge residue that enables a large-scale domain motion of the C-terminal thumb and downstream domains, serving to provide access to the active site cleft, which is largely closed in most experimental polymerase structures in this family. Glycine is commonly found in hinge regions, because of its much greater access to the Ramachandran space, due to its lack of a side chain.

A rotation of the *psi* angle of G809 of the EBOV polymerase from 2° (as in the 7yer.pdb structure) to 40° results in a massive displacement of the upper part of the thumb domains (Figure 12A). It is possible that the nearby P813 may also be involved in fine tuning this motion, as prolines are also involved in hinges, particularly when a restriction on a range of motion is required.

It is notable that, as seen in *Sequence 1* above, near the end of the FS module, there is also a glycine–proline pair, in this case right next to each other, and, with the glycine, one residue past the position of the glycine in the zero-frame protein *Sequence 2*. As shown in Figure 12B, a combination of modest Gly–Pro dihedral angle rotations enables an essentially similar opening of the cleft in the AlphaFold2 model of the dual frameshifted polymerase structure (isoform D). Thus, similar to the conservation of the lysine pair that sticks in towards the active site in both isoforms, the hinge function seems to be equally conserved, despite the change in the protein sequence between the two reading frames. This finding supports the hypothesis that this isoform is functional in a way that is essentially identical to the zero frame L gene product, including the hinge capability, except that it has the added redox module on the back of the polymerase hand.

### 3.8. Genomic Sequences Required for Expression of Frameshift Variants Are Highly Conserved in EBOV

If the predicted FS sites and RdRp isoforms play a significant role in the viral life cycle, they should be highly conserved in EBOV strains, including the FS slippery sequences, sequences required for the DIO2 antisense interaction, and specific coding sequences in the −1 frame, such as the tandem UGA codons and Gly–Pro hinge sequence. Using BLAST searches as described at the end of Methods Section 2.1, several sequence regions critical for FS isoform expression were assessed and found to be essentially 100% conserved in 483 Zaire ebolavirus complete genome samples that were available in the NCBI database at the time (an additional 484th EBOV genome proved to have an incomplete L gene, missing these regions). The sequences searched in this manner and their significance for the predicted L gene FS isoforms are tabulated in Table 1.

Because of the degeneracy of the genetic code, almost every third base has some potential to mutate without altering the RdRp protein sequence encoded by the L gene. Thus, the observed high degree of conservation of specific sequence features such as the potential antisense match to DIO2, and specific codons in the overlapping reading frame, supports the hypothesis that these features are functional. Otherwise, they could easily mutate, given the notoriously mutation-prone nature of RNA viruses.

### 3.9. Tissue Distribution of DIO2 Overlaps with Sites Relevant for Ebola Pathogenesis

Because a complete −1 FS signal exists in the L gene, i.e., the slippery sequence and pseudoknot shown in Figure 2, it is highly likely that truncated FS isoform B can be produced without the participation of DIO2 mRNA. However, with the DIO2 antisense interaction as an alternate trigger for frameshifting that additionally may enable production of the putative selenoprotein module, the possibilities become considerably more complex. Our findings beg the following question: why would the virus evolve an important function that depends upon the presence of a specific cellular mRNA–DIO2–unless an important phase of the virus life cycle and replication is occurring in cells where DIO2 is expressed? As stated in a recent review, DIO2 “is primarily responsible for the local production of T3 inside cells and its presence has been detected in several locations, such as the pituitary gland and hypothalamus, cochlea, brown adipose tissue, bones, muscles, heart, and central nervous system” [45]. Significantly, Ebola virus disease (EVD) is notorious for muscle pain and arthralgia [46], and brown adipose tissue has been identified as a site of infection that may contribute to coagulopathy [47]. There is also abundant evidence of CNS infection in EVD, which may contribute to persistent latent infection [48].

However, the most compelling single piece of evidence of an intimate link between DIO2 and the pathogenesis of EVD lies in the discovery that DIO2 is upregulated in liver during inflammation, by contributing to the innate immune response in macrophages via regulation of intracellular thyroid hormone levels [49]. The DIO2 enzyme activity has also been shown in dendritic cells [50], and DIO2 knockout macrophages show impaired phagocytosis and cytokine responses [51]. This dependence of optimal macrophage function upon intracellular DIO2 expression is hugely significant for EVD, because of the fact that the initial target cells for EBOV upon host entry are dendritic cells and macrophages, which then spread the infection through the body via the circulatory and lymphatic systems, with hepatocytes being one of the primary sites of replication [52]. Because DIO2 in macrophages is upregulated as part of an inflammatory response [49], which often involves increased oxidative stress, it makes sense that the virus might benefit in this environment from the protection afforded by an antioxidant module, expressed in a way that is targeted to a close association with its reactive RNA genetic material (i.e., covalently linked to the viral RdRp).

### 3.10. Redox Biology as a Double-Edged Sword for Both Virus and Host, and the Danger of RNA Oxidation

The multicomponent antioxidant defense systems that exist throughout the kingdoms of life have been viewed in part as a necessary evolutionary development in response to the emergence of oxidative metabolism, which greatly increased the risk of collateral damage by reactive oxygen species (ROS). Antioxidant defenses have also proved essential for compartmentalizing applications of ROS that are beneficial to organisms, particularly their use in defensive immune systems [53]. These applications include things such as the oxidative burst of leukocytes, the degradative mechanisms of phagocytic cells, some mechanisms of cell death induced by killer cells that unleash ROS, and the exploitation of redox signaling in the control of gene expression. Immune activation and inflammation are intimately associated with oxidative stress in many chronic and infectious diseases [54], including many RNA virus infections [55,56,57,58,59,60,61]. In regard to Ebola, a metabolomic study of EVD pathogenesis in humans found evidence of cysteine depletion, suggesting that “oxidative stress, possibly mediated through production of reactive oxygen species by activated neutrophils or macrophages, is a characteristic of EVD regardless of outcome” [62].

Virus-associated oxidative stress can either be a result of host immune activation and inflammation, or a result of viral replication strategies that disrupt cellular redox homeostasis. In most cases, it is very likely a combination of both host and viral factors. Either way, viruses will be subject to harmful consequences of increased ROS, just as their hosts can be, even when it is their own actions that are generating ROS. For viruses like HIV and Ebola, those harms could include lipid peroxidation, affecting the integrity of the viral envelope, and damage to the viral genome via RNA oxidation and free radical attack. This issue brings us back to the following key point made earlier: that RNA oxidation is a major problem for organisms of all types, which have evolved elaborate defense and repair mechanisms to mitigate such damage [43,63].

Examples of viral strategies to mitigate the effects of pro-oxidant environments include virally-encoded glutathione peroxidase genes in HIV-1 and some pox viruses [10,64,65], and, in the case of the Marburg filovirus, the upregulation of Nrf2-mediated cellular antioxidant responses via the viral protein VP24 binding to KEAP1 [66]. In contrast, examples abound of virus-mediated events that increase oxidative stress during infection, many of which are elaborated in the articles cited previously [55,56,57,58,59,60,61].

We have previously provided evidence for a general mechanism by which RNA viruses may contribute to oxidative stress, via the knockdown of host antioxidant enzymes required for DNA synthesis, to conserve the pool of ribonucleotides available for viral replication (i.e., to favor RNA synthesis). This evidence includes computational and experimental evidence of potential antisense targeting of thioredoxin reductase isoforms by HIV-1, EBOV and Zika virus [6,8,29], and for SARS-CoV-2, both RNA mediated and proteolytic knockdown of specific host selenoproteins and antioxidant enzymes [67,68].

Hence, just as for its hosts, EBOV could benefit from localized defense mechanisms, to protect against collateral damage from the oxidative stress resulting from its takeover of host cells. An antioxidant module (e.g., a peroxidase) attached to a protein that binds to the viral mRNA fits that description and could thereby reduce damage to the viral RNA caused by peroxide and other ROS generated from peroxide via the Fenton reaction.

It must also be noted that the EBOV:DIO2 mRNA:mRNA antisense interaction, in addition to the roles proposed here involving frameshifting and SECIS element capture by RNA tethering, could also lead to a decrease in DIO2 protein levels. This result would be consistent with evidence of viral disruptions of selenoprotein synthesis or activity that have now been demonstrated in vitro and in human patients, e.g., for both SARS-CoV-2 [67,68,69,70], and for Zika virus [71,72], for both of which, inter alia, downregulation of selenoprotein P is a biomarker of disease severity.

## 4. Conclusions and Significance

Given that ribosomal frameshifting and translational reprogramming of stop codons are both inherently low efficiency processes, it is highly likely that the major product of a −1 FS at the predicted site would be isoform B, truncated at the first UGA codon in the −1 reading frame. The isoform C product, produced by the minus one FS followed by translational reprogramming of the UGA codons with a downstream stop at proline after 28 residues, would be next in abundance. It could be that formation of isoform B, which only extends through RdRp Motif D, is just the biochemical cost of being able to produce isoform C, which, as shown in Figure 9, adds to the frameshifted equivalent of the Motif E region, essentially completing the polymerase palm domain. Figure 9 also shows that despite the deletion of what is usually called the thumb domain and downstream regions, there is a considerable amount of structure forming what could be considered the base of the thumb that still remains in this truncated form of the enzyme; this structure derives from the N-terminal region of the protein and is clearly visible as a thumb-like projection (red, orange and yellow ribbons) in Figure 9B.

Thus, it is quite possible that, even with truncation essentially at the end of the palm domain, isoform C may be a sufficiently complete RdRp domain to have a limited but functional polymerase activity. As discussed in Section 3.4, it would lack the capping function and methylation activities of native isoform A, but could possibly be capable of significant processive RNA synthesis, and serve a specialized function in replication of the EBOV genome, which is uncapped.

Otherwise, it is possible that isoform C lacks polymerase activity, but may possess sufficient RNA binding ability to be useful for targeting its putative antioxidant module at a location proximal to the viral RNA, thus enhancing its ability to counter RNA oxidation. However, successful addition of the C-terminal domains via the additional execution of the +1 FS of Figure 6 would lead to polymerase isoform D, which is very likely to be a fully-functional RdRp, with the added antioxidant module carried on the back of the polymerase hand, where it would be able to carry out its function in the vicinity of the viral RNA without impinging upon the polymerase related activities of the protein. All of these possibilities regarding potential FS isoforms B–D need to be resolved by experimentation.

We would be remiss if we failed to note several other consequences or possible alternative mechanisms underlying some of our findings. It is very possible that the virus/host antisense interaction shown in Figure 3 could additionally, or even primarily, serve to cause a down-regulation of DIO2, like what we have shown for the Zika virus and selenoprotein P [71]. This interaction could create a more favorable environment for EBOV replication in macrophages. Rather than (or in addition to) ribosomal frameshifting, in the same region of the L gene, there is a potential signal for “transcriptional frameshifting”, a form of RNA editing, by “polymerase stuttering” on the six A bases that encode the pair of lysine residues in the FS region (KK in Figure 9A). That process would be another way of producing a truncated isoform C type polymerase, via a subset of edited mRNA transcripts, or possibly serving as an alternative to the ribosomal +1 FS following the −1 FS, forming isoform D via a different mechanism. Polymerase stuttering on a run of five A bases is the mechanism by which the two alternative forms of the EBOV glycoprotein are formed, so this is an established and plausible viral mechanism [73].

Hopefully, our computational results can provide a blueprint for further validation work by experimentalists. That said, we should note that, if verified experimentally, this would be the first demonstration of internal protein domain swapping at the translational level, achieving a result that normally would require exon shuffling at the RNA level. That this should first be identified in an RNA virus is not surprising, considering that they are constrained by their very small genome size, and thus have few other options to increase their protein coding density other than by using overlapping genes and frameshift related processes.

## Figures and Tables

**Table 1:** BLAST results show a high degree of conservation of FS isoform features in EBOV strains.

EBOV L Gene Sequence Used as BLAST Query	Position in EBOV Genome	Predicted Role in Formation or Function of L Gene FS Isoforms	Percentage of 483 EBOV Strains Showing 100% Identity to Query
*TTTAAAA*CC**TGATGA**AACATTTGTACATT 29-mer	13938-66	−1 FS slippery sequence (*TTTAAAA*), tandem TGA codons and anti-DIO2 antisense sequence	99.8% (482/483) *
C**CGA**TTCTTAACA**CAA**ATGC 20-mer	12531-50	Arg (CGA) and Gln (CAA) codons forming part of peroxiredoxin-like site	100%
GG**G****GTCCA**ATTGCCYCAGTC*CCTTAA* 26-mer Y = C or T, does not affect hinge or frameshift residues	14005-30	Hinge residue Gly 809 in zero frame (GGG), Gly–Pro in −1 frame (**GGTCCA**) and +1 FS hexamer (*CCTTAA*)	100% 74% Y = C 26% Y = T

* In one of 483 EBOV complete genomes, just the first base of the 29-mer shown had mutated to a C.

## Data Availability

All data used in this study are included in the text and Supplementary Materials of the article.

## Supplementary Materials

The following supporting information can be downloaded at: https://www.mdpi.com/article/10.3390/pathogens13100829/s1, File S1: EBOVpolymeraseFSisoformModels.prj. Figure S1: Identification of potential -1 frameshift site in the Zaire ebolavirus L gene using Knotinframe with default search parameters, as reported previously (Taylor 2014). Figure S2. RNA structure prediction for region past the putative +1 FS sequence CCUUAA. Figure S3. RNA structure prediction for internal base pairings in DIO and EBV oligos used in gel mobility shift assay: explanation for very faint labeling of EBV oligo relative to DIO oligo. Table S1. Protein sequences used to build AlphaFold2 models of the predicted EBOV L gene frameshift isoforms C and D (Fasta format). Table S2. Averaged data from 3 independent experiments (A–C) involving transfection of the dual reporter +1 frameshift construct, as graphed in Figure 7. Table S3. Comparison of inter-residue distances for key residues of peroxiredoxin 1 active site to redox domain encoded as −1 frameshift isoform of EBOV L gene RdRp. Ref. [74] was cited in Supplementary Materials.

## References

[1] Report of an International Commission (1978). Ebola Haemorrhagic Fever in Zaire: 1976. Bull. World Health Organ..

[2] Letafati A., Salahi Ardekani O., Karami H., Soleimani M. (2023). Ebola virus disease: A narrative review. Microb. Pathog..

[3] Sanjuán R., Nebot M.R., Chirico N., Mansky L.M., Belshaw R. (2010). Viral Mutation Rates. J. Virol..

[4] Feldmann H., Klenk H.D. (1996). Marburg and Ebola Viruses. Adv. Virus Res..

[5] Whelan S.P.J., Barr J.N., Wertz G.W. (2004). Transcription and Replication of Nonsegmented Negative-Strand RNA Viruses. Curr. Top. Microbiol. Immunol..

[6] Taylor E.W., Ruzicka J.A., Premadasa L., Zhao L. (2016). Cellular Selenoprotein mRNA Tethering via Antisense Interactions with Ebola and HIV-1 mRNAs May Impact Host Selenium Biochemistry. Curr. Top. Med. Chem..

[7] Taylor E.W., Ruzicka J.A., Preamadasa L. Translational Readthrough of the Ebola Nucleoprotein 3’-UGA Codon via Antisense Tethering of Thioredoxin Reductase 3 mRNA. Proceedings of the International Congress on Targeting Ebola.

[8] Premadasa L., Dailey G., Ruzicka J.A., Taylor E.W. (2021). Selenium-Dependent Read Through of the Conserved 3’-Terminal UGA Stop Codon of HIV-1 nef. Am. J. Biopharm. Pharm. Sci..

[9] Taylor E.W., Cox A.G., Zhao L., Ruzicka J.A., Bhat A.A., Zhang W., Nadimpalli R.G., Dean R.G. (2000). Nutrition, HIV, and Drug Abuse: The Molecular Basis of a Unique Role for Selenium. J. Acquir. Immune Defic. Syndr..

[10] Zhao L., Cox A.G., Ruzicka J.A., Bhat A.A., Zhang W., Taylor E.W. (2000). Molecular Modeling and in vitro Activity of an HIV-1-Encoded Glutathione Peroxidase. Proc. Natl. Acad. Sci. USA.

[11] Zhang W., Ramanathan C.S., Nadimpalli R.G., Bhat A.A., Cox A.G., Taylor E.W. (1999). Selenium-Dependent Glutathione Peroxidase Modules Encoded by RNA Viruses. Biol. Trace Elem. Res..

[12] Volchkov V.E., Volchkova V.A., Chepurnov A.A., Blinov V.M., Dolnik O., Netesov S.V., Feldmann H. (1999). Characterization of the L Gene and 5h Trailer Region of Ebola Virus. J. Gen. Virol..

[13] Jacks T., Varmus H.E. (1985). Expression of the Rous Sarcoma Virus *Pol* Gene by Ribosomal Frameshifting. Science.

[14] Jacks T., Power M.D., Masiarz F.R., Luciw P.A., Barr P.J., Varmus H.E. (1988). Characterization of Ribosomal Frameshifting in HIV-1 Gag-Pol Expression. Nature.

[15] Choi J., O’Loughlin S., Atkins J.F., Puglisi J.D. (2020). The Energy Landscape of −1 Ribosomal Frameshifting. Sci. Adv..

[16] Olubajo B., Taylor E.W. (2005). A −1 Frameshift in the HIV-1 Env Gene Is Enhanced by Arginine Deficiency via a Hungry Codon Mechanism. Mutat. Res..

[17] Cao S., Chen S.-J. (2008). Predicting Ribosomal Frameshifting Efficiency. Phys. Biol..

[18] Green L., Kim C.-H., Bustamante C., Tinoco I. (2008). Characterization of the Mechanical Unfolding of RNA Pseudoknots. J. Mol. Biol..

[19] Lin Z., Gilbert R.J.C., Brierley I. (2012). Spacer-Length Dependence of Programmed −1 or −2 Ribosomal Frameshifting on a U6A Heptamer Supports a Role for Messenger RNA (mRNA) Tension in Frameshifting. Nucleic Acids Res..

[20] Taylor E.W. (2014). Sequence Analysis Reveals a −1 Ribosomal Frameshift Site in the Zaire Ebolavirus Polymerase Gene in a Region with Antisense Complementarity to a Human Selenoprotein mRNA. Figshare.

[21] Rodnina M.V., Korniy N., Klimova M., Karki P., Peng B.-Z., Senyushkina T., Belardinelli R., Maracci C., Wohlgemuth I., Samatova E. (2020). Translational Recoding: Canonical Translation Mechanisms Reinterpreted. Nucleic Acids Res..

[22] Atkins J.F., Loughran G., Bhatt P.R., Firth A.E., Baranov P.V. (2016). Ribosomal Frameshifting and Transcriptional Slippage: From Genetic Steganography and Cryptography to Adventitious Use. Nucleic Acids Res..

[23] Advani V.M., Dinman J.D. (2016). Reprogramming the Genetic Code: The Emerging Role of Ribosomal Frameshifting in Regulating Cellular Gene Expression. BioEssays.

[24] Dinman J.D., Ruiz-Echevarria M.J., Peltz S.W. (1998). Translating Old Drugs into New Treatments: Ribosomal Frameshifting as a Target for Antiviral Agents. Trends Biotechnol..

[25] Firth A.E., Brierley I. (2012). Non-Canonical Translation in RNA Viruses. J. Gen. Virol..

[26] Theis C., Reeder J., Giegerich R. (2008). KnotInFrame: Prediction of −1 Ribosomal Frameshift Events. Nucleic Acids Res..

[27] Skalsky R.L., Cullen B.R. (2010). Viruses, microRNAs, and Host Interactions. Annu. Rev. Microbiol..

[28] Lei L., Cheng A., Wang M., Jia R. (2022). The Influence of Host miRNA Binding to RNA Within RNA Viruses on Virus Multiplication. Front. Cell. Infect. Microbiol..

[29] Taylor E.W. (2020). RNA Viruses vs. DNA Synthesis: A General Viral Strategy That May Contribute to the Protective Antiviral Effects of Selenium.

[30] Howard M.T., Gesteland R.F., Atkins J.F. (2004). Efficient Stimulation of Site-Specific Ribosome Frameshifting by Antisense Oligonucleotides. RNA.

[31] Henderson C.M., Anderson C.B., Howard M.T. (2006). Antisense-Induced Ribosomal Frameshifting. Nucleic Acids Res..

[32] Ishimaru D., Plant E.P., Sims A.C., Yount B.L., Roth B.M., Eldho N.V., Pérez-Alvarado G.C., Armbruster D.W., Baric R.S., Dinman J.D. (2013). RNA Dimerization Plays a Role in Ribosomal Frameshifting of the SARS Coronavirus. Nucleic Acids Res..

[33] De Smit M.H., Van Duin J., Van Knippenberg P.H., Van Eijk H.G. (1994). CCC.UGA: A New Site of Ribosomal Frameshifting in Escherichia Coli. Gene.

[34] Jumper J., Evans R., Pritzel A., Green T., Figurnov M., Ronneberger O., Tunyasuvunakool K., Bates R., Žídek A., Potapenko A. (2021). Highly Accurate Protein Structure Prediction with AlphaFold. Nature.

[35] Rehmsmeier M., Steffen P., Hochsmann M., Giegerich R. (2004). Fast and Effective Prediction of microRNA/Target Duplexes. RNA.

[36] Grentzmann G., Ingram J.A., Kelly P.J., Gesteland R.F., Atkins J.F. (1998). A Dual-Luciferase Reporter System for Studying Recoding Signals. RNA.

[37] Mirdita M., Schütze K., Moriwaki Y., Heo L., Ovchinnikov S., Steinegger M. (2022). ColabFold-Making Protein Folding Accessible to All. Nat. Methods.

[38] Liang B. (2020). Structures of the *Mononegavirales* Polymerases. J. Virol..

[39] Tchesnokov E.P., Raeisimakiani P., Ngure M., Marchant D., Götte M. (2018). Recombinant RNA-Dependent RNA Polymerase Complex of Ebola Virus. Sci. Rep..

[40] Gladyshev V.N., Jeang K.T., Stadtman T.C. (1996). Selenocysteine, Identified as the Penultimate C-Terminal Residue in Human T-Cell Thioredoxin Reductase, Corresponds to TGA in the Human Placental Gene. Proc. Natl. Acad. Sci. USA.

[41] Shimodaira S., Iwaoka M. (2019). Synthesis of Selenocysteine-Containing Dipeptides Modeling the Active Site of Thioredoxin Reductase. Phosphorus Sulfur Silicon Relat. Elem..

[42] Xu X.-M., Turanov A.A., Carlson B.A., Yoo M.-H., Everley R.A., Nandakumar R., Sorokina I., Gygi S.P., Gladyshev V.N., Hatfield D.L. (2010). Targeted Insertion of Cysteine by Decoding UGA Codons with Mammalian Selenocysteine Machinery. Proc. Natl. Acad. Sci. USA.

[43] Li Z., Malla S., Shin B., Li J.M. (2014). Battle against RNA Oxidation: Molecular Mechanisms for Reducing Oxidized RNA to Protect Cells. WIREs RNA.

[44] Nelson K.J., Knutson S.T., Soito L., Klomsiri C., Poole L.B., Fetrow J.S. (2011). Analysis of the Peroxiredoxin Family: Using Active-site Structure and Sequence Information for Global Classification and Residue Analysis. Proteins Struct. Funct. Bioinforma..

[45] Sabatino L., Vassalle C., Del Seppia C., Iervasi G. (2021). Deiodinases and the Three Types of Thyroid Hormone Deiodination Reactions. Endocrinol. Metab..

[46] Amissah-Arthur M.B., Poller B., Tunbridge A., Adebajo A. (2018). Musculoskeletal Manifestations of Ebola Virus. Rheumatology.

[47] Gourronc F.A., Rebagliati M.R., Kramer-Riesberg B., Fleck A.M., Patten J.J., Geohegan-Barek K., Messingham K.N., Davey R.A., Maury W., Klingelhutz A.J. (2022). Adipocytes Are Susceptible to Ebola Virus Infection. Virology.

[48] Billioux B.J., Smith B., Nath A. (2016). Neurological Complications of Ebola Virus Infection. Neurotherapeutics.

[49] Kwakkel J., Surovtseva O.V., De Vries E.M., Stap J., Fliers E., Boelen A. (2014). A Novel Role for the Thyroid Hormone-Activating Enzyme Type 2 Deiodinase in the Inflammatory Response of Macrophages. Endocrinology.

[50] Van Der Spek A.H., Fliers E., Boelen A. (2017). Thyroid Hormone Metabolism in Innate Immune Cells. J. Endocrinol..

[51] Van Der Spek A.H., Surovtseva O.V., Jim K.K., Van Oudenaren A., Brouwer M.C., Vandenbroucke-Grauls C.M.J.E., Leenen P.J.M., Van De Beek D., Hernandez A., Fliers E. (2018). Regulation of Intracellular Triiodothyronine Is Essential for Optimal Macrophage Function. Endocrinology.

[52] Mahanty S., Bray M. (2004). Pathogenesis of Filoviral Haemorrhagic Fevers. Lancet Infect. Dis..

[53] Martinvalet D., Walch M. (2022). Editorial: The Role of Reactive Oxygen Species in Protective Immunity. Front. Immunol..

[54] Biswas S.K. (2016). Does the Interdependence between Oxidative Stress and Inflammation Explain the Antioxidant Paradox?. Oxid. Med. Cell. Longev..

[55] Schwarz K.B. (1996). Oxidative Stress during Viral Infection: A Review. Free Radic. Biol. Med..

[56] Herzenberg L.A., De Rosa S.C., Dubs J.G., Roederer M., Anderson M.T., Ela S.W., Deresinski S.C., Herzenberg L.A. (1997). Glutathione Deficiency Is Associated with Impaired Survival in HIV Disease. Proc. Natl. Acad. Sci. USA.

[57] Taylor E.W. (2010). The Oxidative Stress-Induced Niacin Sink (OSINS) Model for HIV Pathogenesis. Toxicology.

[58] Khomich O.A., Kochetkov S.N., Bartosch B., Ivanov A.V. (2018). Redox Biology of Respiratory Viral Infections. Viruses.

[59] Liu M., Chen F., Liu T., Chen F., Liu S., Yang J. (2017). The Role of Oxidative Stress in Influenza Virus Infection. Microbes Infect..

[60] Dobrzyńska M., Moniuszko-Malinowska A., Skrzydlewska E. (2023). Metabolic Response to CNS Infection with Flaviviruses. J. Neuroinflamm..

[61] Georgieva E., Ananiev J., Yovchev Y., Arabadzhiev G., Abrashev H., Abrasheva D., Atanasov V., Kostandieva R., Mitev M., Petkova-Parlapanska K. (2023). COVID-19 Complications: Oxidative Stress, Inflammation, and Mitochondrial and Endothelial Dysfunction. Int. J. Mol. Sci..

[62] Eisfeld A.J., Halfmann P.J., Wendler J.P., Kyle J.E., Burnum-Johnson K.E., Peralta Z., Maemura T., Walters K.B., Watanabe T., Fukuyama S. (2017). Multi-Platform ’Omics Analysis of Human Ebola Virus Disease Pathogenesis. Cell Host Microbe.

[63] Kong Q., Lin C.G. (2010). Oxidative Damage to RNA: Mechanisms, Consequences, and Diseases. Cell. Mol. Life Sci..

[64] Senkevich T.G., Bugert J.J., Sisler J.R., Koonin E.V., Darai G., Moss B. (1996). Genome Sequence of a Human Tumorigenic Poxvirus: Prediction of Specific Host Response-Evasion Genes. Science.

[65] Bratke K.A., McLysaght A. (2008). Identification of Multiple Independent Horizontal Gene Transfers into Poxviruses Using a Comparative Genomics Approach. BMC Evol. Biol..

[66] Edwards M.R., Johnson B., Mire C.E., Xu W., Shabman R.S., Speller L.N., Leung D.W., Geisbert T.W., Amarasinghe G.K., Basler C.F. (2014). The Marburg Virus VP24 Protein Interacts with Keap1 to Activate the Cytoprotective Antioxidant Response Pathway. Cell Rep..

[67] Wang Y., Huang J., Sun Y., Stubbs D., He J., Li W., Wang F., Liu Z., Ruzicka J.A., Taylor E.W. (2021). SARS-CoV-2 Suppresses mRNA Expression of Selenoproteins Associated with Ferroptosis, Endoplasmic Reticulum Stress and DNA Synthesis. Food Chem. Toxicol..

[68] Gallardo I.A., Todd D.A., Lima S.T., Chekan J.R., Chiu N.H., Taylor E.W. (2023). SARS-CoV-2 Main Protease Targets Host Selenoproteins and Glutathione Biosynthesis for Knockdown via Proteolysis, Potentially Disrupting the Thioredoxin and Glutaredoxin Redox Cycles. Antioxidants.

[69] Moghaddam A., Heller R., Sun Q., Seelig J., Cherkezov A., Seibert L., Hackler J., Seemann P., Diegmann J., Pilz M. (2020). Selenium Deficiency Is Associated with Mortality Risk from COVID-19. Nutrients.

[70] Rayman M.P., Taylor E.W., Zhang J. (2022). The Relevance of Selenium to Viral Disease with Special Reference to SARS-CoV-2 and COVID-19. Proc. Nutr. Soc..

[71] Dailey G.P., Premadasa L.S., Ruzicka J.A., Taylor E.W. (2021). Inhibition of Selenoprotein Synthesis by Zika Virus May Contribute to Congenital Zika Syndrome and Microcephaly by Mimicking SELENOP Knockout and the Genetic Disease PCCA. BBA Adv..

[72] Sosa-Acosta P., Quiñones-Vega M., Guedes J.D.S., Rocha D., Guida L., Vasconcelos Z., Nogueira F.C.S., Domont G.B. (2024). Multiomics Approach Reveals Serum Biomarker Candidates for Congenital Zika Syndrome. J. Proteome Res..

[73] Sanchez A.M., Trappier S.G., Mahy B.W., Peters C.J., Nichol S.T. (1996). The virion glycoproteins of Ebola viruses are encoded in two reading frames and are expressed through transcriptional editing. Proc. Natl. Acad. Sci. USA.

[74] Yuan B., Peng Q., Cheng J., Wang M., Zhong J., Qi J., Gao G.F., Shi Y. (2022). Structure of the Ebola Virus Polymerase Complex. Nature.

