# Supplementary material for: Modular Polymerase Synthesis and Internal Protein Domain Swapping via Dual Opposed Frameshifts in the Ebola Virus L Gene
Source: Pathogens. 2024 Sep 25;13(10):829. doi: 10.3390/pathogens13100829 (PMC11510084; doi:10.3390/pathogens13100829)
Supplement: Supplementary file 1 [file pathogens-13-00829-s001.zip › Supplemental material Stubbs et al 2024.zip!/Supplemental material Stubbs et al 2024.pdf]

Supplemental Material for Stubbs et al. 2024

Figure S1. Identification of potential -1 frameshift site in the Zaire ebolavirus L gene using Knotinframe [1] with default search parameters, as reported previously (Taylor 2014 [2]).

```
>Ebola L gene fragment
  Rank:               1
  Slippery sequence: tttaaaa
  Slippery position: 338
  Substring length:  60
  Deltarel:          0.052
  345  cctgatgaaacatttgtacattcaggttttatctattttggaaaaaaacaatatttgaat  405
-9.60  ...[[[[[[[[.........{{{{{]]]]]]]]....(((........)))...}}}}}.  knotted structure
```

Figure S2. RNA structure prediction for region past the putative +1 FS sequence CCUUAA

The sequence shown here begins at CCUUAA. The first stem-loop at left is that shown as A:A' in Figure 6, in which the potential pseudoknotted structure with B:B' may be part of a more extended structure involving the second stem-loop shown here starting at residue 48. The B:B' interaction could involve "kissing loops" between the 2 stem-loops, involving residues numbered 31-33 pairing with 63-61, as connected below. Figure generated via RNAstructure 6.5 using default parameters (https://rna.urmc.rochester.edu/RNAstructure.html).

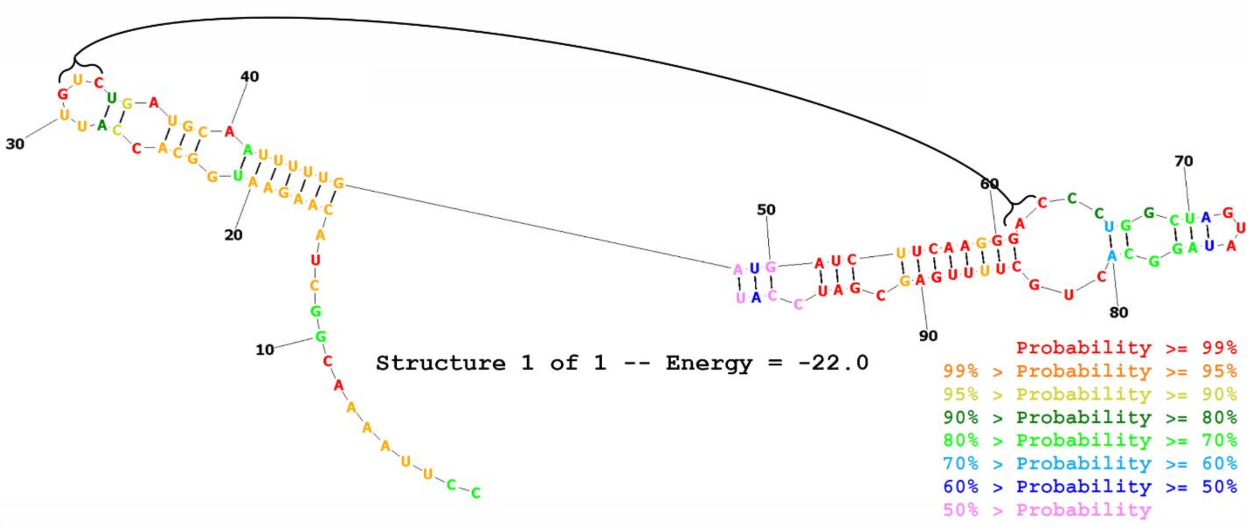

Figure S3. RNA structure prediction for internal base pairings in DIO and EBV oligos used in gel mobility shift assay: explanation for very faint labeling of EBV oligo relative to DIO oligo

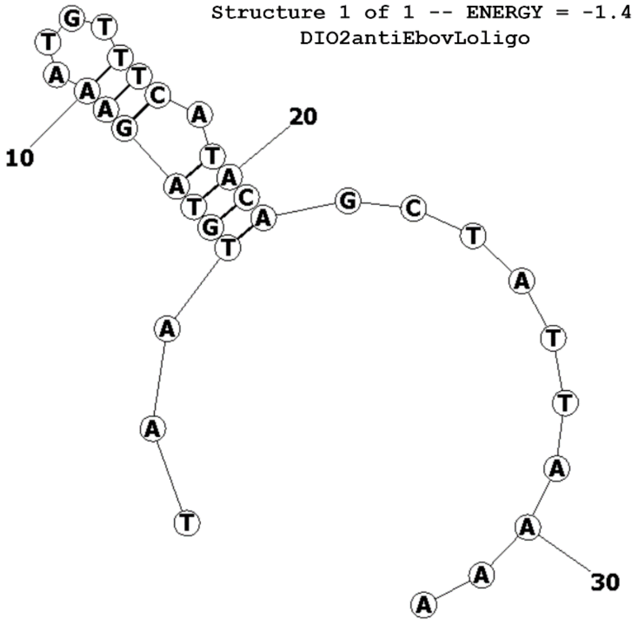

Ethidium bromide labels nucleic acids by acting as an intercalating agent. Thus, labeling of single-stranded oligonucleotides depends upon the presence of internal secondary structure, i.e. base paired regions. Folding of the DNA oligo labeled DIO in Figure 5 gives this predicted structure, containing a 4 base pair stem involving the A base at position 20. This base is on an unpaired bulge in the DIO-EBV hybrid duplex shown in Figure 4; thus, the complementary EBV oligo is unable to make a similar stem. In contrast, folding of the EBV oligo results in the outcome "The given structure contains no pairs", explaining why the EBV oligo is so weakly labelled in the gel shift experiment (Figure 5). This figure was generated via RNAstructure 6.5 using default parameters (https://rna.urmc.rochester.edu/RNAstructure.html).

Table S1. Protein sequences used to build AlphaFold2 models [3,4] of the predicted EBOV L gene frameshift isoforms C and D (Fasta format)

>EBOV RdRp FS isoform C
PDARLSSPIVLDQCDLVTRACGLYSSYSLNPQLRNCKLPKHIYRLKYDVTVTKFLSDVPVATLPIDFIVPVLLKALS
GNGFCPVEPRCQQFLDEIIKYTMQDALFLKYYLKNVGAQEDCVDEHFQEKILSSIQGNEFLHQMFFWYDLAIL
TRRGRLNRGNSRSTWFVHDDLIDILGYGDYVFWKIPISMLPLNTQGIPHAAMDWYQASVFKEAVQGHTHIV
SVSTADVLIMCKDLITCRFNTTLISKIAEIEDPVCSDYPNFKIVSMLYQSGDYLLSILGSDGYKIIKFLEPLCLAKIQL
CSKYTERKGRFLTQMHLAVNHTLEEITEMRALKPSQAQKIREFHRTLIRLEMTPQQLCELFSIQKHWGHPVLH
SETAIQKVKKHATVLKALRPIVIFETYCVFKYSIAKHYFDSQGSWYSVTSDRNLTPGLNSYIKRNQFPPLPMIKEL
LWEFYHLDHPPLFSTKIISDLSIFIKDRATAVERTCWDAVFEPNVLGYNPPHKFSTKRVPEQFLEQENFSIENVL
SYAQKLEYLLPQYRNFSFSLKEKELNVGRTFGKLPYPTRNVQTLCEALLADGLAKAFPSNMMVVTEREQKESL
LHQASWHHTSDDFGEHATVRGSSFVTDLEKYNLAFRYEFTAPFIEYCNRCYGVKNVFNWMHYTIPQCYMH
VSDYYNPPHNLTLENRDNPPEGPSSYRGHMGGIEGLQQKLWTSISCAQISLVEIKTGFKLRSAVMGDNQCIT
VLSVFPLETDADEQEQSAEDNAARVAASLAKVTSACGIFLKTUUNICTFRFYLFWKKTIFEWGPIASVP

In the EBOV RdRp protein refseq file (NP_066251), this sequence begins at residue 9, as that is the N-terminal of the comparison experimental structure (7YER.pdb [5]). The sequence shown was used in its entirety to build the AlphaFold2 model shown in Figure 9. The frameshift module encoded in the -1 reading frame is highlighted. The two underlined Sec codons (U) were altered to C for the Alphafold job, then mutated to Sec as a final step.

>EBOV RdRp FS isoform D
PDARLSSPIVLDQCDLVTRACGLYSSYSLNPQLRNCKLPKHIYRLKYDVTVTKFLSDVPVATLPIDFIVPVLLKALS
GNGFCPVEPRCQQFLDEIIKYTMQDALFLKYYLKNVGAQEDCVDEHFQEKILSSIQGNEFLHQMFFWYDLAIL
TRRGRLNRGNSRSTWFVHDDLIDILGYGDYVFWKIPISMLPLNTQGIPHAAMDWYQASVFKEAVQGHTHIV
SVSTADVLIMCKDLITCRFNTTLISKIAEIEDPVCSDYPNFKIVSMLYQSGDYLLSILGSDGYKIIKFLEPLCLAKIQL
CSKYTERKGRFLTQMHLAVNHTLEEITEMRALKPSQAQKIREFHRTLIRLEMTPQQLCELFSIQKHWGHPVLH
SETAIQKVKKHATVLKALRPIVIFETYCVFKYSIAKHYFDSQGSWYSVTSDRNLTPGLNSYIKRNQFPPLPMIKEL
LWEFYHLDHPPLFSTKIISDLSIFIKDRATAVERTCWDAVFEPNVLGYNPPHKFSTKRVPEQFLEQENFSIENVL
SYAQKLEYLLPQYRNFSFSLKEKELNVGRTFGKLPYPTRNVQTLCEALLADGLAKAFPSNMMVVTEREQKESL
LHQASWHHTSDDFGEHATVRGSSFVTDLEKYNLAFRYEFTAPFIEYCNRCYGVKNVFNWMHYTIPQCYMH
VSDYYNPPHNLTLENRDNPPEGPSSYRGHMGGIEGLQQKLWTSISCAQISLVEIKTGFKLRSAVMGDNQCIT
VLSVFPLETDADEQEQSAEDNAARVAASLAKVTSACGIFLKTUUNICTFRFYLFWKKTIFEWGPIASVPKTATR
MAPLSDAIFDDLQGTLASIGTAFERSISETRHIFPCRITAAFHTFFSVRILQYHHLGFNKGFDLGQLTLGKPLDFG
TISLALAVPQVLGGLSFLNPEKCFYRNLGDPVTSGLFQLKTYLRMIEMDDLFLPLIAKNPGNCTAIDFVLNPSGL
NVPGSQDLTSFLRQIVRRTITLSAKNKLINTLFHASADFEDEMVCKWLLSSTPVMSRFAADIFSRTPSGKRLQIL
GYLEGTRTLLASKIINNNTETPVLDRLRKITLQRWSLWFSYLDHCDNILAEALTQITCTVDLAQILREYSWAHILE
GRPLIGATLPCMIEQFKVFWLKPYEQCPQCSNAKQPGGKPFVSVAVKKHIVSAWPNASRISWTIGDGIPYIGS
RTEDKIGQPAIKPKCPSAALREAIELASRLTWVTQGSSNSDLLIKPFLEARVNLSVQEILQMTPSHYSGNIVHRY
NDQYSPHSFMANRMSNSATRLIVSTNTLGEFSGGGQSARDSNIIFQNVINYAVALFDIKFRNTEATDIQYNRA
HLHLTKCCTREVPAQYLTYTSTLDLDLTRYRENELIYDS

In the EBOV RdRp protein refseq file (NP_066251), this sequence begins at residue 9 and only extends to residue 1383, as that is where the comparison experimental structure (7YER.pdb [5]) terminates, i.e., omitting C terminal domains 1384-2212 of the L protein. The sequence shown was used in its entirety to build the AlphaFold2 model shown in Figure 11B. The frameshift module encoded in the -1 reading frame is highlighted. The two underlined Sec codons (U) were altered to C for the Alphafold job, then mutated to Sec as a final step.

Table S2. Averaged data from 3 independent experiments (A-C) involving transfection of the dual reporter +1 frameshift construct, as graphed in Figure 7.

Exp	N	Wildtype	SD Wildtype	Mutant	SD Mutant	Zero-L	SD Zero-L
A	5	0.1974	0.0410	1	0.281	0.0883	0.0199
B	6	0.1554	0.0320	1	0.250	0.0585	0.0182
C	6	0.1491	0.0119	1	0.116	0.0640	0.0160

A-C averaged results:

	Wildtype	Mutant	Zero-LUC
Avg	0.1673	1	0.07027
SD	0.02626	0.2274	0.01586
SEM	0.01516	0.1313	0.00916
N	*3*	*3*	*3*

Unpaired T-tests:

WT vs Zero-LUC *p = 0.0054*

WT vs MUTANT *p = 0.0032*

Mut vs ZERO-LUC *p = 0.0021*

Note: The results are expressed as the ratio of Luciferase/GFP signals and then normalized to Mutant = 1 because, with both reporter genes in the same reading frame, that construct was designed to give a 1:1 molar ratio of LUC:GFP, assuming ribosomal transit of the entire mRNA.

Table S3. Comparison of inter-residue distances for key residues of peroxiredoxin 1 active site to redox domain encoded as -1 frameshift isoform of EBOV L gene RdRp

Peroxiredoxin active site residue pair	Distance between sidechain β-carbons in 1PRX.PDB	Distance between sidechain β-carbons in EBOV FS domain	Δ*d*
Threonine to cysteine (or SeCys in EBOV)	4.6 Å	5.2 Å	0.6 Å
Threonine to arginine	13.3 Å	12.1 Å	1.2 Å
Arginine to cysteine (or SeCys in EBOV)	9.7 Å	8.8 Å	0.9 Å
Arginine to glutamate (or Gln in EBOV)	5.6 Å	6.0 Å	0.4 Å

See Figure 11 for visual representation of the geometry of this active site
Average Δ*d* < 0.8 Å, less than 10% of the average C_{β}-C_{β} distance in the peroxiredoxin structure

References for Supplemental Material

1. Theis, C.; Reeder, J.; Giegerich, R. KnotInFrame: Prediction of -1 Ribosomal Frameshift Events. *Nucleic Acids Res.* **2008**, *36*, 6013–6020, doi:10.1093/nar/gkn578.
2. Taylor, E.W. Sequence Analysis Reveals a -1 Ribosomal Frameshift Site in the Zaire Ebolavirus Polymerase Gene in a Region with Antisense Complementarity to a Human Selenoprotein mRNA (Preprint). **2014**, https://doi.org/10.6084/m9.figshare.24081828.v1
3. Jumper, J.; Evans, R.; Pritzel, A.; Green, T.; Figurnov, M.; Ronneberger, O.; Tunyasuvunakool, K.; Bates, R.; Žídek, A.; Potapenko, A.; et al. Highly Accurate Protein Structure Prediction with AlphaFold. *Nature* **2021**, *596*, 583–589, doi:10.1038/s41586-021-03819-2.
4. Mirdita, M.; Schütze, K.; Moriwaki, Y.; Heo, L.; Ovchinnikov, S.; Steinegger, M. ColabFold - Making Protein Folding Accessible to All. (Preprint). **2021,** http://biorxiv.org/lookup/doi/10.1101/2021.08.15.456425.
5. Yuan, B.; Peng, Q.; Cheng, J.; Wang, M.; Zhong, J.; Qi, J.; Gao, G.F.; Shi, Y. Structure of the Ebola Virus Polymerase Complex. *Nature* **2022**, *610*, 394–401, doi:10.1038/s41586-022-05271-2.
